# Open-access quantitative MRI data of the spinal cord and reproducibility across participants, sites and manufacturers

**DOI:** 10.1038/s41597-021-00941-8

**Published:** 2021-08-16

**Authors:** Julien Cohen-Adad, Eva Alonso-Ortiz, Mihael Abramovic, Carina Arneitz, Nicole Atcheson, Laura Barlow, Robert L. Barry, Markus Barth, Marco Battiston, Christian Büchel, Matthew Budde, Virginie Callot, Anna J. E. Combes, Benjamin De Leener, Maxime Descoteaux, Paulo Loureiro de Sousa, Marek Dostál, Julien Doyon, Adam Dvorak, Falk Eippert, Karla R. Epperson, Kevin S. Epperson, Patrick Freund, Jürgen Finsterbusch, Alexandru Foias, Michela Fratini, Issei Fukunaga, Claudia A. M. Gandini Wheeler-Kingshott, Giancarlo Germani, Guillaume Gilbert, Federico Giove, Charley Gros, Francesco Grussu, Akifumi Hagiwara, Pierre-Gilles Henry, Tomáš Horák, Masaaki Hori, James Joers, Kouhei Kamiya, Haleh Karbasforoushan, Miloš Keřkovský, Ali Khatibi, Joo-Won Kim, Nawal Kinany, Hagen H. Kitzler, Shannon Kolind, Yazhuo Kong, Petr Kudlička, Paul Kuntke, Nyoman D. Kurniawan, Slawomir Kusmia, René Labounek, Maria Marcella Laganà, Cornelia Laule, Christine S. Law, Christophe Lenglet, Tobias Leutritz, Yaou Liu, Sara Llufriu, Sean Mackey, Eloy Martinez-Heras, Loan Mattera, Igor Nestrasil, Kristin P. O’Grady, Nico Papinutto, Daniel Papp, Deborah Pareto, Todd B. Parrish, Anna Pichiecchio, Ferran Prados, Àlex Rovira, Marc J. Ruitenberg, Rebecca S. Samson, Giovanni Savini, Maryam Seif, Alan C. Seifert, Alex K. Smith, Seth A. Smith, Zachary A. Smith, Elisabeth Solana, Y. Suzuki, George Tackley, Alexandra Tinnermann, Jan Valošek, Dimitri Van De Ville, Marios C. Yiannakas, Kenneth A. Weber II, Nikolaus Weiskopf, Richard G. Wise, Patrik O. Wyss, Junqian Xu

**Affiliations:** 1 NeuroPoly Lab, Institute of Biomedical Engineering, Polytechnique Montreal, Montreal, QC Canada; 2 Functional Neuroimaging Unit, CRIUGM, Université de Montréal, Montreal, QC Canada; 3 Mila - Quebec AI Institute, Montreal, QC Canada; 4 Department of Radiology, Swiss Paraplegic Centre, Nottwil, Switzerland; 5 Centre for Advanced Imaging, The University of Queensland, Brisbane, Australia; 6 Department of Radiology, University of British Columbia, Vancouver, BC Canada; 7 Athinoula A. Martinos Center for Biomedical Imaging, Department of Radiology, Massachusetts General Hospital, Charlestown, MA USA; 8 Department of Radiology, Harvard Medical School, Boston, MA USA; 9 Harvard–Massachusetts Institute of Technology Health Sciences & Technology, Cambridge, MA USA; 10 School of Information Technology and Electrical Engineering, The University of Queensland, Brisbane, Australia; 11 NMR Research Unit, Queen Square MS Centre, UCL Queen Square Institute of Neurology, Faculty of Brain Sciences, University College London, London, UK; 12 Department of Systems Neuroscience, University Medical Center Hamburg-Eppendorf, Hamburg, Germany; 13 Department of Neurosurgery, Medical College of Wisconsin, Milwaukee, WI USA; 14 Aix-Marseille Univ, CNRS, CRMBM, Marseille, France; 15 APHM, Hopital Universitaire Timone, CEMEREM, Marseille, France; 16 Vanderbilt University Institute of Imaging Science, Vanderbilt University Medical Center, Nashville, TN USA; 17 Department of Computer and Software Engineering, Polytechnique Montreal, Montreal, Canada; 18 CHU Sainte-Justine Research Centre, Montreal, QC Canada; 19 Centre de Recherche CHUS, CIMS, Sherbrooke, Canada; 20 Sherbrooke Connectivity Imaging Lab (SCIL), Computer Science department, Université de Sherbrooke, Sherbrooke, Canada; 21 Université de Strasbourg, CNRS, ICube, Strasbourg, France; 22 UHB - University Hospital Brno and Masaryk University, Department of Radiology and Nuclear Medicine, Brno, Czech Republic; 23 McConnell Brain Imaging Centre, Montreal Neurological Institute, McGill University, Montreal, QC Canada; 24 Department of Physics and Astronomy, University of British Columbia, Vancouver, BC Canada; 25 Max Planck Institute for Human Cognitive and Brain Sciences, Leipzig, Germany; 26 Richard M. Lucas Center, Stanford University School of Medicine, Stanford, CA USA; 27 Spinal Cord Injury Center Balgrist, University of Zurich, Zurich, Switzerland; 28 Institute of Nanotechnology, CNR, Rome, Italy; 29 IRCCS Santa Lucia Foundation, Rome, Italy; 30 Department of Radiology, Juntendo University School of Medicine, Tokyo, Japan; 31 Department of Brain and Behavioural Sciences, University of Pavia, Pavia, Italy; 32 Brain MRI 3T Research Centre, IRCCS Mondino Foundation, Pavia, Italy; 33 MR Clinical Science, Philips Healthcare, Markham, ON Canada; 34 CREF - Museo storico della fisica e Centro studi e ricerche Enrico Fermi, Rome, Italy; 35 Radiomics Group, Vall d’Hebron Institute of Oncology, Vall d’Hebron Barcelona Hospital Campus, Barcelona, Spain; 36 Center for Magnetic Resonance Research, Department of Radiology, University of Minnesota, Minneapolis, MN USA; 37 Multimodal and functional imaging laboratory, Central European Institute of Technology (CEITEC), Brno, Czech Republic; 38 Department of Radiology, Toho University Omori Medical Center, Tokyo, Japan; 39 Department of Radiology, the University of Tokyo, Tokyo, Japan; 40 Interdepartmental Neuroscience Program, Feinberg School of Medicine, Northwestern University, Chicago, IL USA; 41 Department of Psychiatry and Behavioral Sciences, School of Medicine, Stanford University, Stanford, CA USA; 42 Centre of Precision Rehabilitation for Spinal Pain (CPR Spine), School of Sport, Exercise and Rehabilitation Sciences, College of Life and Environmental Sciences, University of Birmingham, Edgbaston, Birmingham, UK; 43 BioMedical Engineering and Imaging Institute (BMEII), Department of Radiology, Icahn School of Medicine at Mount Sinai, New York, NY USA; 44 Institute of Bioengineering/Center for Neuroprosthetics, Ecole Polytechnique Fédérale de Lausanne, Geneva, Switzerland; 45 Department of Radiology and Medical Informatics, University of Geneva, Geneva, Switzerland; 46 Institute of Diagnostic and Interventional Neuroradiology, Carl Gustav Carus University Hospital, Technische Universität Dresden, Dresden, Germany; 47 Department Of Medicine (Neurology), University of British Columbia, Vancouver, BC Canada; 48 CAS Key Laboratory of Behavioral Science, Institute of Psychology, Chinese Academy of Sciences, Beijing, China; 49 Department of Psychology, University of Chinese Academy of Sciences, Beijing, China; 50 Wellcome Centre For Integrative Neuroimaging, FMRIB, Nuffield Department of Clinical Neurosciences, University of Oxford, Oxford, UK; 51 CUBRIC, Cardiff University, Wales, UK; 52 Centre for Medical Image Computing (CMIC), Medical Physics and Biomedical Engineering Department, University College London, London, UK; 53 Epilepsy Society MRI Unit, Chalfont St Peter, UK; 54 Division of Clinical Behavioral Neuroscience, Department of Pediatrics, University of Minnesota, Minneapolis, MN USA; 55 Departments of Neurology and Biomedical Engineering, University Hospital Olomouc, Olomouc, Czech Republic; 56 IRCCS Fondazione Don Carlo Gnocchi ONLUS, Milan, Italy; 57 Departments of Radiology, Pathology & Laboratory Medicine, Physics & Astronomy; International Collaboration on Repair Discoveries (ICORD), University of British Columbia, Vancouver, BC Canada; 58 Division of Pain Medicine, Department of Anesthesiology, Perioperative and Pain Medicine, Stanford University School of Medicine, Stanford, CA USA; 59 Department of Neurophysics, Max Planck Institute for Human Cognitive and Brain Sciences, Leipzig, Germany; 60 Department of Radiology, Beijing Tiantan Hospital, Capital Medical University, Beijing, China; 61 Tiantan Image Research Center, China National Clinical Research Center for Neurological Diseases, Beijing, China; 62 Center of Neuroimmunology, Laboratory of Advanced Imaging in Neuroimmunological Diseases, Hospital Clinic Barcelona, Institut d’Investigacions Biomèdiques August Pi i Sunyer (IDIBAPS) and Universitat de Barcelona, Barcelona, Spain; 63 Fondation Campus Biotech Genève, 1202 Geneva, Switzerland; 64 Department of Radiology, Vanderbilt University Medical Center, Nashville, TN USA; 65 UCSF Weill Institute for Neurosciences, Department of Neurology, University of California San Francisco, San Francisco, CA USA; 66 Neuroradiology Section, Vall d’Hebron University Hospital, Barcelona, Spain; 67 E-health Centre, Universitat Oberta de Catalunya, Barcelona, Spain; 68 School of Biomedical Sciences, Faculty of Medicine, The University of Queensland, Brisbane, Australia; 69 University of Oklahoma Health Sciences Center, Oklahoma City, OK USA; 70 Department of Neurology, Faculty of Medicine and Dentistry, Palacký University and University Hospital Olomouc, Olomouc, Czech Republic; 71 Felix Bloch Institute for Solid State Physics, Faculty of Physics and Earth Sciences, Leipzig University, Leipzig, Germany; 72 Institute for Advanced Biomedical Technologies, Department of Neuroscience, Imaging and Clinical Sciences, “G. D’Annunzio University” of Chieti-Pescara, Chieti-Pescara, Italy

**Keywords:** Imaging techniques, Spinal cord diseases, Databases

## Abstract

In a companion paper by Cohen-Adad *et al*. we introduce the *spine generic* quantitative MRI protocol that provides valuable metrics for assessing spinal cord macrostructural and microstructural integrity. This protocol was used to acquire a single subject dataset across 19 centers and a multi-subject dataset across 42 centers (for a total of 260 participants), spanning the three main MRI manufacturers: GE, Philips and Siemens. Both datasets are publicly available via git-annex. Data were analysed using the Spinal Cord Toolbox to produce normative values as well as inter/intra-site and inter/intra-manufacturer statistics. Reproducibility for the *spine generic* protocol was high across sites and manufacturers, with an average inter-site coefficient of variation of less than 5% for all the metrics. Full documentation and results can be found at https://spine-generic.rtfd.io/. The datasets and analysis pipeline will help pave the way towards accessible and reproducible quantitative MRI in the spinal cord.

## Abstract (summary)

**Table:** 

Measurement(s)	spinal cord
Technology Type(s)	magnetic resonance imaging
Factor Type(s)	manufacturer • site
Sample Characteristic - Organism	Homo sapiens
Sample Characteristic - Location	Canada • Switzerland • Australia • United States of America • United Kingdom • Germany • French Republic • Czech Republic • Italy • Japan • Kingdom of Spain • China

Machine-accessible metadata file describing the reported data: 10.6084/m9.figshare.14052269

## Background & Summary

Quantitative MRI (qMRI) aims at providing objective continuous metrics that specifically reflect the morphology, microstructure and/or chemical composition of tissues^1,2^, thereby enabling deeper insight and understanding of disease pathophysiology. While qMRI techniques have been successfully implemented in the brain for several decades, they remain largely underutilized for spinal cord (SC) imaging in both clinical and research settings, mostly as a direct consequence of the many challenges that need to be overcome in order to acquire good quality data^3,4^. In a companion paper^5^, we introduce the *spine generic* protocol for acquiring high-quality qMRI of the human SC at 3 Tesla (T). The *spine generic* protocol includes relevant sequences and contrasts for calculating metrics sensitive to macrostructural and microstructural integrity: T1w and T2w imaging for SC cross-sectional area (CSA) computation, multi-echo gradient echo for gray matter CSA, as well as magnetization transfer and diffusion weighted imaging for assessing white matter microstructure.

To demonstrate the practical implementation and reproducibility of the *spine generic* protocol, single subject and multi-subject datasets were acquired across multiple centers. Relevant qMRI metrics were calculated using a fully-automatic analysis pipeline, and those metrics were compared within site, across sites (within manufacturer), and across different manufacturers. The generated normative values will be useful as reference for future clinical studies.

## Methods

### Data acquisition

Single-participant and multi-participant datasets were acquired across multiple centers (see details below). The scan operator (researcher or MR technician) was instructed to follow the *spine-generic* protocol^5^. Briefly, each participant was positioned in the head-first supine position. The following sequences were run: localizer, 3D sagittal T1w, 3D sagittal T2w, 2D axial diffusion weighted echo planar imaging (30 directions at a b-value of 800 s/mm^2^), 3D axial gradient echo with/without an MT pulse and an additional T1w scan, 2D axial multi-echo gradient echo. Data were collected, organized and analyzed according to a fully-documented procedure available at https://spine-generic.rtfd.io.

### Ethical compliance

We have complied with all relevant ethical regulations. Local ethics committees of the participating institutions listed in Online-only Table 1 approved the study protocol. Signed informed consent was obtained from all participants under the compliance of the corresponding local ethics committee and stored in the corresponding local research center under responsibility of the local principal investigator/s (listed as *“Contact”* in the Online-only Table 1).

### Single subject

The same participant (JCA, 38 y.o., male) was scanned at 19 different centers within 77 days. This represents a “best case scenario” in terms of reproducibility because the participant was very familiar with being scanned, knew how to position himself in the scanner and how to breathe, directly interacted with the MR technician at each site to ensure the standard operating procedure (SOP) was understood, and was able to adapt the sequence parameters on the scanner if the protocol was not imported properly (e.g., hardware or software version incompatibility).

### Multiple subjects

In order to evaluate a more realistic (routine) scenario, 42 different groups worldwide with varying levels of expertise each scanned six different healthy participants (3 males, 3 females), aged between 19 and 56 y.o., median age 28 y.o., resulting in 260 data sets. Participant-specific list of age and sex distribution is available at https://github.com/spine-generic/data-multi-subject/blob/r20201130/participants.tsv. Each group used the *spine generic* protocol and SOP, and obtained consent to scan and upload participants’ anonymized data onto a publicly-available repository. Anatomical scans (T1w, T2w) where facial features are visible were “defaced” before being released to the public domain using *pydeface*^6^. Some centers equipped with more than one system were asked to scan 6 different participants on each scanner, although they were also given the possibility to scan the same 6 participants across systems for convenience. In the latter case, this could slightly bias the inter-site coefficient of variation (COV), however it would not affect the inter-manufacturer COV. The list of centers and scanner models is shown in Online-only Table 1.

Data from each participant was then entered into the processing pipeline described in the next section. COVs were computed within site and manufacturers, and compared across manufacturers.

### Data processing

**Fig. 1:**
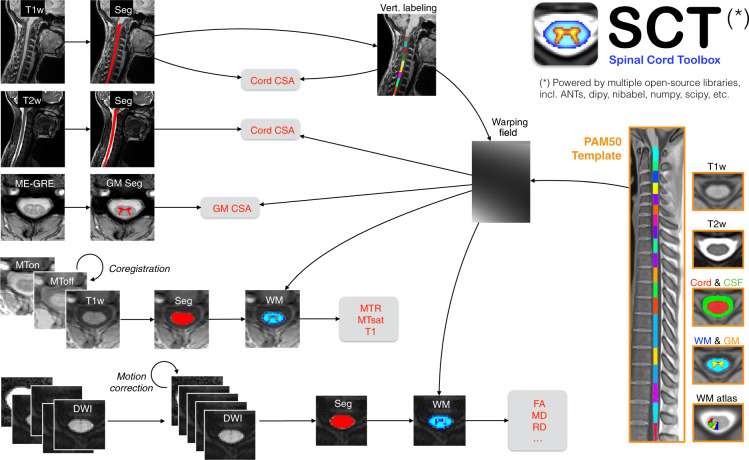
Overview of the processing pipeline based on SCT. Briefly, for each participant, the SC is automatically segmented on the T1w, T2w, GRE-T1w, and mean DWI scans, while the gray matter is segmented on the ME-GRE scan (after averaging across echoes). Vertebral labeling is run on the T1w scan, followed by registration of the PAM50 template to each contrast. Estimated metrics are shown in red.

Data were processed using Spinal Cord Toolbox (SCT) v5.0.1^7^ and spine-generic v2.6 (https://github.com/spine-generic/spine-generic/releases/tag/v2.6). The processing pipeline is illustrated in Fig. 1 and is fully documented at https://spine-generic.rtfd.io. Briefly, for the T1w scan, the SC was segmented using deep-learning^8^, vertebral levels were identified^9^ and the SC was registered to the PAM50 template^10^ using the C3 and C5 vertebrae as labels. Then, the SC CSA was computed slice-wise (corrected for angulation between the SC and the slice) and averaged between the C2 and C3 vertebral levels. For the T2w scan, the SC was segmented^8^, registered with the PAM50 template using the transformation from the T1w scan and the CSA was computed and averaged between C2 and C3. For the ME-GRE (T2*w) scan, the SC^8^ and gray matter (GM)^11^ were segmented and registered with the PAM50 template using the transformation from the T1w scan. Then, GM CSA was computed and averaged between C3 and C4. For the magnetization transfer (MT) protocol, the SC on the GRE-T1w scan was segmented^8^ and registered with the PAM50 template using the initial transformation from the T1w scan. GRE-MT1 and GRE-MT0 scans were registered to the GRE-T1w scans using an automatically-generated mask tightly fitting the SC for more accurate registration. Magnetization transfer ratio (MTR) and MTsat^12^ were computed and their values extracted for the white matter (WM) between C2 and C5 using the WM probabilistic atlas^13^. For the diffusion weighted imaging (DWI) scan, the time series of diffusion-weighted scans were averaged and the SC was segmented so that a mask could be created around it. The time series were motion-corrected using slice-wise 2D transformations regularized along z^7^, with time-adjacent volumes grouped together for increased robustness^14^. The PAM50 template was then registered to the DWI dataset using the initial transformation from the T1w scan and diffusion tensor imaging (DTI) metrics were computed using the weighted least squares fitting algorithm implemented in Dipy^15^ and wrapped in SCT’s function sct_dmri_compute_dti. No post-processing was performed. Lastly, DTI metrics were extracted within the WM between C2 and C5.

Processing was run on a supercomputer cluster (https://docs.computecanada.ca/wiki/Graham), by distributing 32x OpenMP jobs across 9 nodes in parallel (each node equipped with 2 x Intel E5-2683 v4 Broadwell @ 2.1 GHz, with 128GB reserved RAM), enabling us to process all 260 participants in parallel (one participant per CPU core). Software parallelization was achieved using Python’s *multiprocessing* package available in SCT’s sct_run_batch. Total processing times were 37 min (single subject) and 40 min (multi-subject).

### Statistics

Intra- and inter-manufacturer differences were tested using a one-way ANOVA. Post-hoc analyses testing pairwise differences across manufacturers were performed using the Tukey Honestly Significant Difference (HSD) using the family-wise error rate to account for multiple comparisons. Significance level was set to p = 0.05. Interactive plots available online were generated with Plotly (https://plotly.com/).

## Data Records

The two datasets associated with this publication are:

- The Spine Generic Public Database (Single Subject)^16^
- The Spine Generic Public Database (Multi-Subject)^17^

### Dataset management

**Fig. 2:**
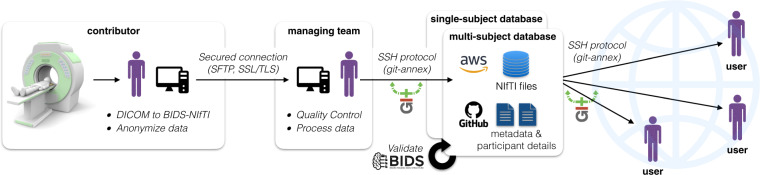
Illustration of the dataset management, from acquisition to end-user consumption.

Figure 2 illustrates the data management workflow. Datasets are managed using git-annex (https://git-annex.branchable.com/); git-annex is built on git technology and enables the separation of large files (NIfTI images, hosted on Amazon Web Services, AWS) from small files (metadata and documentation, hosted on GitHub). This decision was based on the modularity of git-annex (multiple mirrors can be added) and its compatibility with Datalad^18^. The documentation for contributing to the repositories is hosted on a wiki (https://github.com/spine-generic/spine-generic/wiki).

To facilitate data aggregation across centers, we used the *Brain Imaging Data Structure* (BIDS) convention^19^. BIDS notably features JSON metadata files as a sidecar for each NIfTI file, which includes relevant acquisition parameters, making it easy to assess how well each site followed the generic protocol and which parameters were modified. Parameter verification (within a specified tolerance) as well as file and folder naming is automated (https://spine-generic.rtfd.io/en/latest/data-acquisition.html#checking-acquisition-parameters) such that, every time new participants are added to the database, a notification is sent to a continuous integration system (e.g., https://github.com/spine-generic/data-multi-subject/runs/2730553396?check_suite_focus=true) that downloads the dataset and runs custom scripts to verify the validity of the dataset. For example, if a flip angle for a particular volume exceeded the tolerance range, the BIDS validator would fail and the data would not be merged. In that case, the management team would reach out to the data contributors asking if they can reacquire the data. If not, the data would not be added to the dataset. Another (less problematic) example: if a file was incorrectly named (sub_amu01_T2w.nii.gz instead of sub-amu01_T2w.nii.gz), the BIDS validator would fail. In that case, the management team would manually correct the file name, commit and push the change to the working branch and wait for the BIDS validator to pass before being able to merge the new data on the main (master) branch. Below is an example of the “BIDS Validator” script output (only showing part of it):


WARNING: Incorrect FlipAngle: sub-amu01_T2w.nii.gz; FA=180 instead of 120WARNING: Incorrect RepetitionTime: sub-amu02_T2w.nii.gz; TR=2 instead of 1.5WARNING: Incorrect FlipAngle: sub-amu02_T2w.nii.gz; FA=180 instead of 120WARNING: Incorrect RepetitionTime: sub-amu03_T2w.nii.gz; TR=2 instead of 1.5WARNING: Incorrect FlipAngle: sub-amu03_T2w.nii.gz; FA=135 instead of 120Missing jsonSidecar:./derivatives/labels/sub-oxfordOhba05/anat/sub-oxfordOhba05_acq-T1w_MTS_seg-manual.jsonMissing jsonSidecar:./derivatives/labels/sub-oxfordOhba05/anat/sub-oxfordOhba05_T1w_labels-disc-manual.jsonMissing jsonSidecar:./derivatives/labels/sub-oxfordOhba05/anat/sub-oxfordOhba05_T1w_RPI_r_labels-manual.json


## Technical Validation

### Data quality

**Fig. 3:**
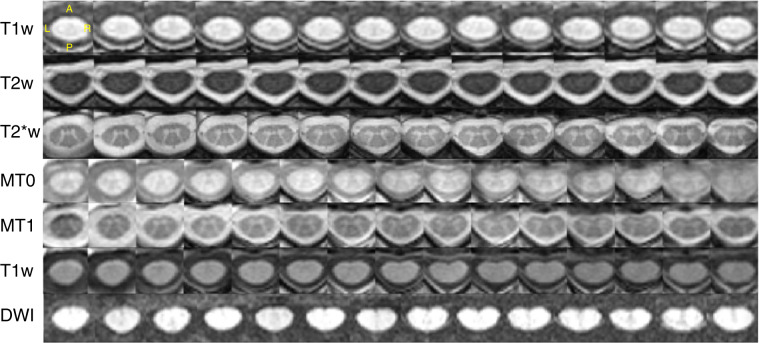
Axial views of good quality data for all sequences in the spine generic protocol across various slices (the exact coverage along the SC varies because the slice thickness varies across sequences). DWI corresponds to the mean DWI data after motion correction. The images are from different participants. T1w: *vuiisAchieva02*; T2w: *milan01*; T2*w (ME-GRE): *brnoCeitec01*; MT0, MT1, T1w (for the MTS protocol) and DWI : *barcelona04*. Axial views were automatically generated by SCT’s QC report.

Overall, data quality was satisfactory based on qualitative visual inspection. Criteria included the correctness of field of view prescription, proper selection of receive coils, quality of shimming (assessed by looking at fat saturation performance and the presence of susceptibility distortions), and the presence and severity of motion artifacts. Figure 3 shows examples of good quality data for all sequences. A few operator errors occurred, including: mis-labeled MT0 for MT1 and MT1 for MT0, shim parameters changed between MT0 and MT1 scans (causing different signal intensities, and hence not suitable for MT-based metrics), change of FFT scaling factor between the MT1/MT0 scans and the T1w scan used to compute MTsat and T1 maps (causing different signal quantization and hence not suitable for MT-based metrics unless corrected for), and repositioning of the participants, causing mis-alignment between the images before/after repositioning and violation of the analysis pipeline assumptions (all images are supposed to be acquired with the patient in the same position). These errors were not caught by the BIDS validator, but by the managing team during visual inspection of the data and interpretation of the qMRI metrics results. In future work, the data validator could be made sensitive to these issues. For example, the FFT scaling factor and shim coefficient are sometimes retrievable from the DICOM data and could be checked. Also, the qform (affine matrix present in the NIfTI header) could be checked to ensure consistency across data from the same series, e.g. MT1, MT0, GRE-T1w. Regarding the mis-labeling of MT1/MT0, training a deep learning model to recognize image contrast could address this issue.

**Fig. 4:**
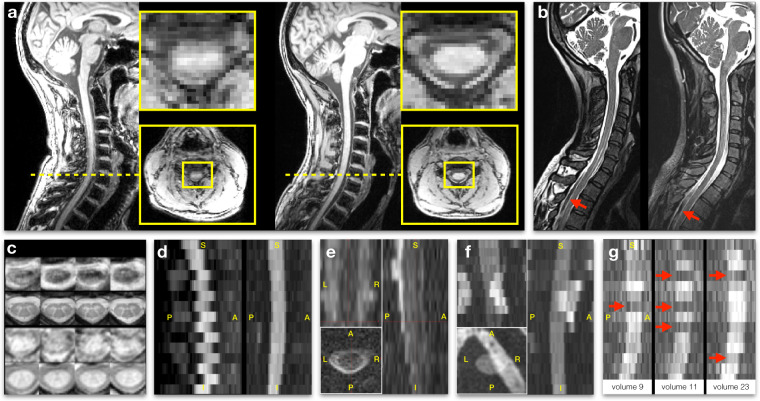
Examples of image artifacts: (**a**) T1w MPRAGE taken in the same participant (from the single subject database) at two different sites on a Siemens Prisma: oxfordFmrib (left) and juntendoPrisma (right). The slightly larger cervical lordosis on the left likely induced more pronounced cerebrospinal fluid (CSF) flow and SC motion resulting in the artifact shown in the axial view. (**b**) T2w scans showing signal drops in the CSF likely due to a poorly-recovered CSF signal combined with flow effects. These two participants (beijingVerio01 and strasbourg03), were acquired with a flip angle of 180° instead of the recommended 120°, which likely explained the presence of those artifacts (longer TR was required for sufficient T1 recovery). (**c**) Axial view of ME-GRE scans with (fslAchieva04, 1st row) and without motion (brnoCeitec01, 2nd row), and axial view of GRE-MT0 with (fslAchieva04, 3rd row) and without motion (barcelona04, 4th row). (**d**) Mean DWI scan from a Philips site (ubc02, left panel) with a concatenated acquisition wherein odd slices are acquired during the first half of the entire acquisition (spanning all b-vectors) and the even slices are acquired during the second half. In the event of participant motion between those two acquisition sub-sets, apparent motion will be visible between the odd and even slices. When odd and even slices are acquired closer in time (in ascending/descending mode, or interleaved but sequentially within the same b-vector), this artifact is not visible (mountSinai03, right panel). Such an artifact could be problematic for image registration with regularization along the S-I axis, or for performing diffusion tractography. (**e**) b=0 image from a DWI scan (perform02) acquired with poor shimming and resulting signal dropout. (**f**) Another example of poor shimming resulting in sub-efficient fat saturation, with the fat being aliased on top of the SC. Here we show the mean DWI scan of a participant from the single subject database (perform). (**g**) Effect of pulsatile movement on a non-cardiac gated acquisition (single subject, juntendoAchieva). Diffusion-weighted scans (sagittal view) acquired at three b-vecs fairly orthogonal to the SC (i.e., diffusion-specific signal attenuation should be minimum in the SC), showing abrupt signal drop at a few slices (red arrows), likely due to cardiac-related pulsatile effects.

Figure 4 illustrates some of the image artifacts encountered during QC. A list of poor data quality scans is available on the Github’s issues of the dataset under the label “data-quality” (https://github.com/spine-generic/data-multi-subject/labels/data-quality); most of these were caused by patient motion. Mosaics of images for every contrast and every participant are available in the supplementary materials (Figures S1–S5). Additional examples of good quality data are also available in the spine generic website (https://spine-generic.rtfd.io/en/latest/data-acquisition.html#example-of-datasets).

### Quantitative results: Single subject

Overall, data quality was satisfactory. All images were visually inspected to ensure that there were no significant errors in the masks used to average the signals in the SC, WM or GM, and any errors were manually corrected. A list of poor quality scans is available on Github in the issues for the dataset, under the label “data-quality” (https://github.com/spine-generic/data-single-subject/labels/data-quality). Complete metrics and statistical tests are available in the r20201130 release assets (https://github.com/spine-generic/data-single-subject/releases/download/r20201130/results.zip).

**Fig. 5:**
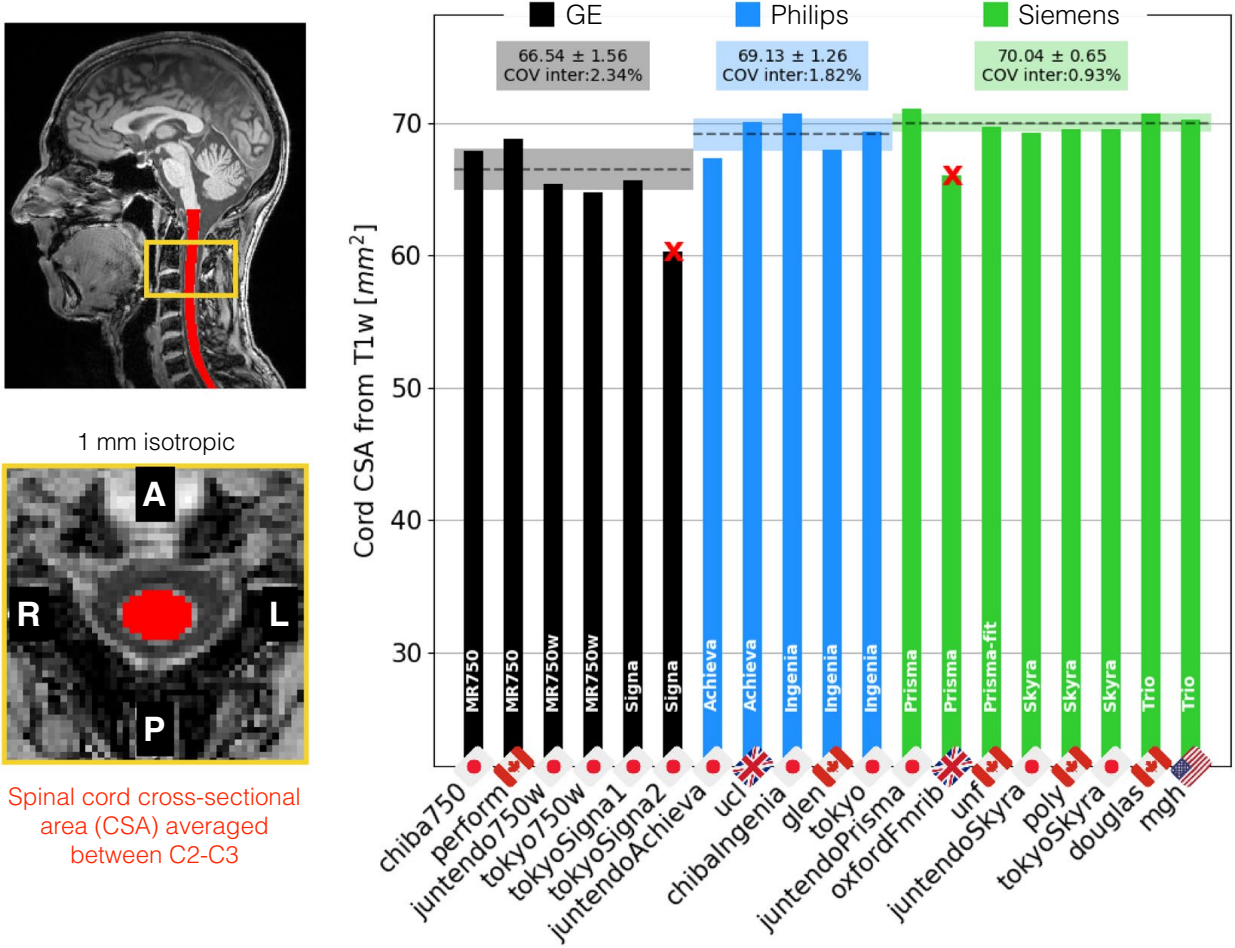
Results of the single subject study for the T1w scan. The cross-sectional area (CSA) of the SC was averaged between the C2 and C3 vertebral levels. Sites *tokyoSigna2* and *oxfordFmrib* were excluded from the statistics due to excessive motion.

Figure 5 shows the SC CSA data from the T1w scan, averaged between cervical levels 2 and 3 (C2 and C3), for the single participant across the 19 centers. Within each manufacturer, the inter-site standard deviation ranges from 0.65 mm^2^ (Siemens) to 1.56 mm^2^ (GE), which is remarkably small considering that the size of a pixel is 1 mm^2^. The inter-site COVs were 2.3% for GE, 1.8% for Philips and 0.9% for Siemens. The inter-manufacturer difference was significant (p < 0.01), with the Tukey test showing significant differences between GE and Philips (p-adjusted = 0.03) and between GE and Siemens (p-adjusted < 0.01).

**Fig. 6:**
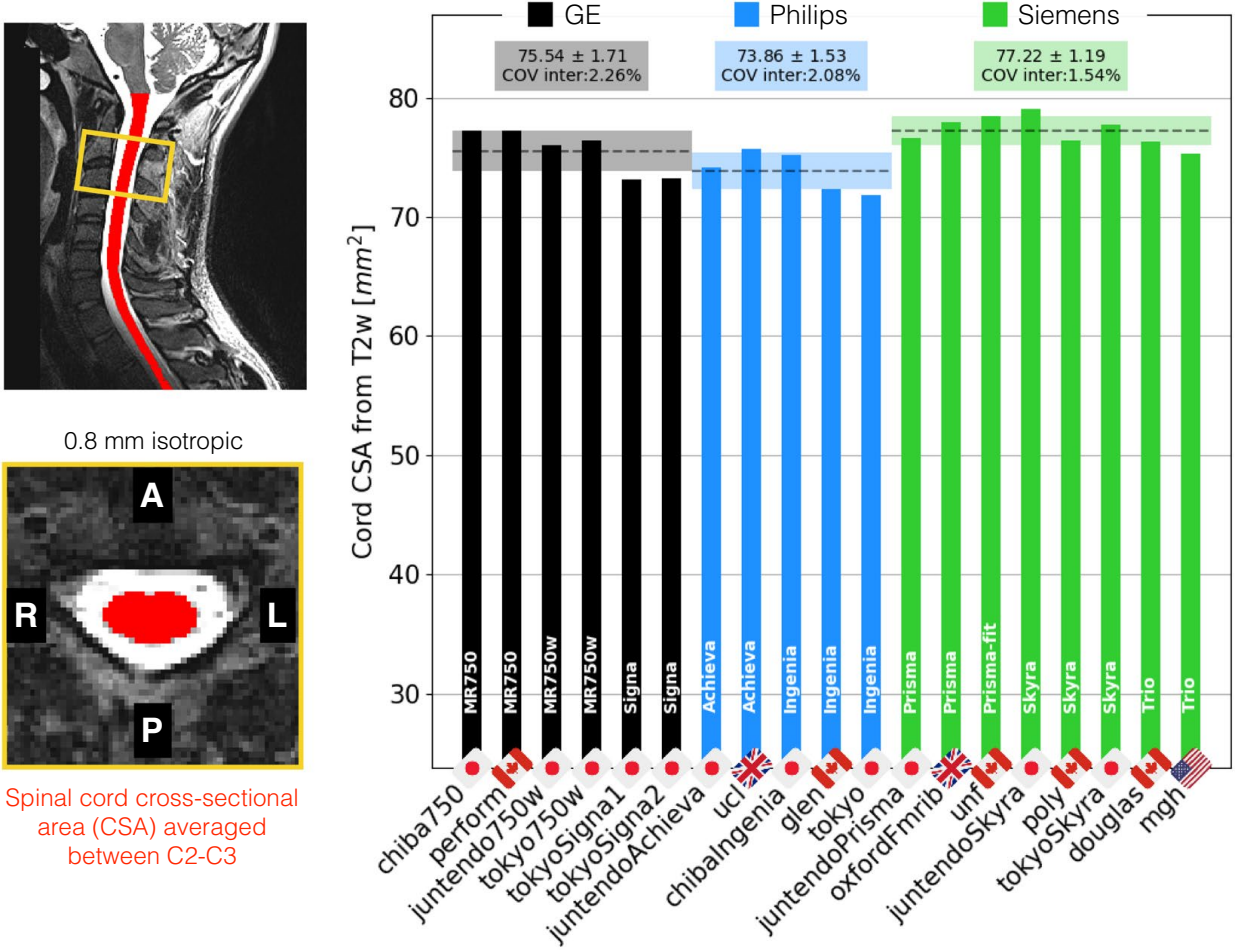
Results of the single subject study for the T2w scan. The cross-sectional area (CSA) of the SC was averaged between the C2 and C3 vertebral levels.

Figure 6 shows the SC CSA for the T2w scan, again averaged between cervical levels 2 and 3 (C2 and C3). The inter-site COVs were 2.3% for GE, 2.1% for Philips and 1.5% for Siemens. The inter-manufacturer difference was significant (p < 0.01), with the Tukey test showing significant differences between Philips and Siemens (p-adjusted < 0.01).

**Fig. 7:**
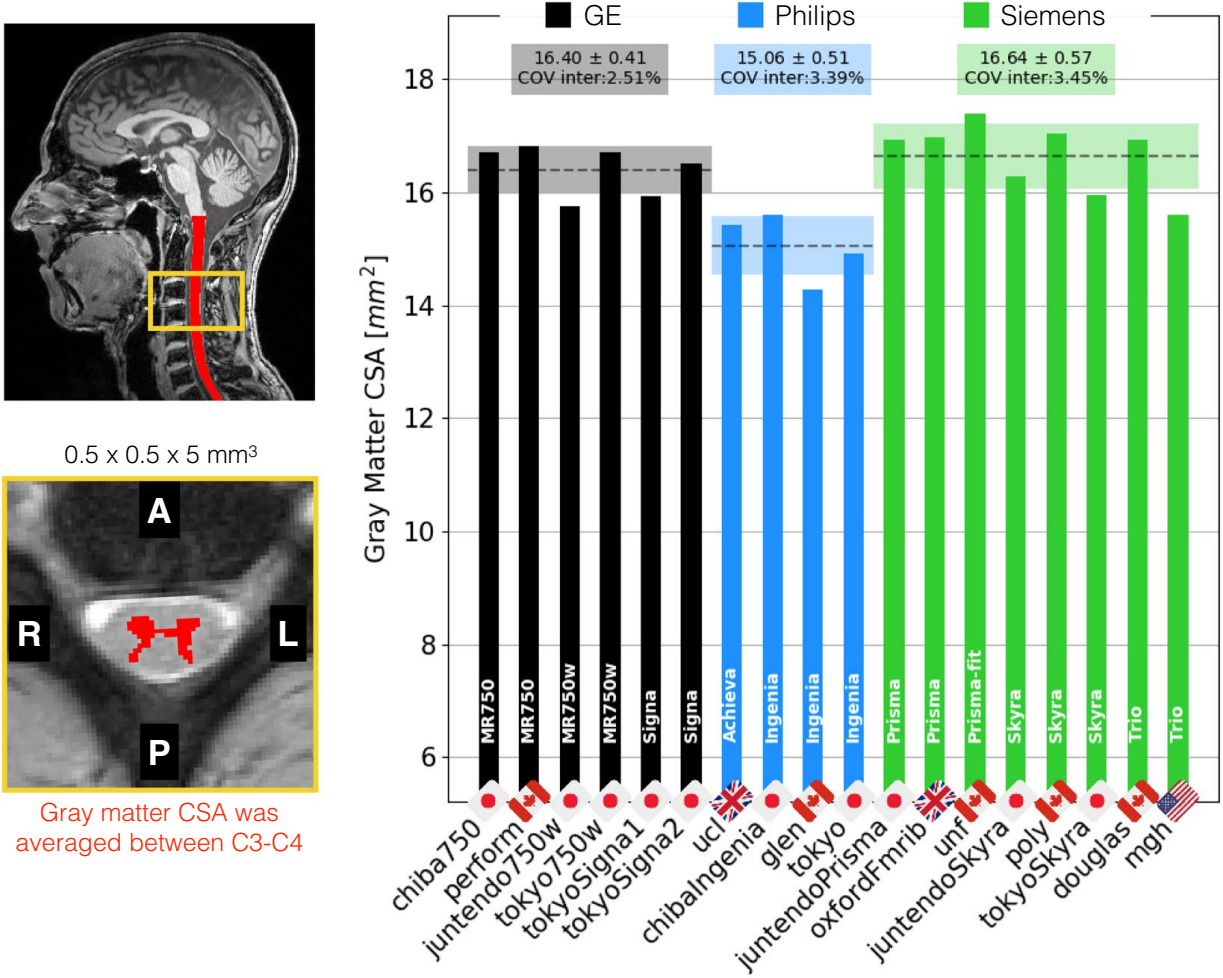
Results of the single subject study for the ME-GRE scan. Gray matter CSA was computed after automatic gray matter segmentation and averaged between C3 and C4 vertebral levels.

Figure 7 shows the gray matter CSA for the ME-GRE scan, averaged between cervical levels C3 and C4. The inter-site COVs were 2.5% for GE, 3.4% for Philips and 3.4% for Siemens. The inter-manufacturer difference was significant (p < 0.01), with the Tukey test showing significant differences between GE and Philips (p-adjusted < 0.01) and between Philips and Siemens (p-adjusted < 0.01).

**Fig. 8:**
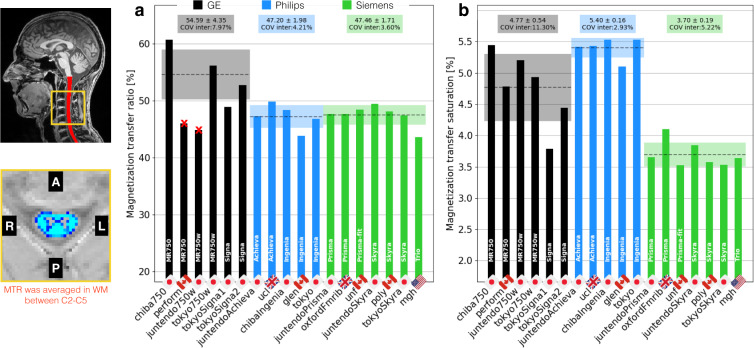
Results of the single subject study for the MT protocol. The mean MTR (**a**) and MTsat (**b**) were computed in the white matter between C2 and C5. Sites *perform* and *juntendo750w* were excluded from the statistics because the TR for the GRE-MT0 and GRE-MT1 was set to 62 ms (vs. 35ms for the other GE sites), causing drastic decrease of MTR values. These sites were not excluded from MTsat.

Figure 8a shows the MTR average for the WM between C2 and C5. The inter-site COVs were 8.0% for GE, 4.2% for Philips and 3.6% for Siemens. The inter-manufacturer difference was significant (p < 0.01), but the Tukey test showed no significant difference across pairwised manufacturers.

Figure 8b shows the MTsat results. The inter-site COVs were 11.3% for GE, 2.9% for Philips and 5.2% for Siemens. The inter-manufacturer difference was significant (p < 0.01), with the Tukey test showing significant differences between GE and Philips (p-adjusted = 0.03), between GE and Siemens (p-adjusted < 0.01), and between Philips and Siemens (p-adjusted < 0.01).

Sites *perform* and *juntendo750w* were excluded from the MTR statistics because the TR for the GRE-MT0 and GRE-MT1 was set to 62 ms (vs. 35 ms for the other GE sites), causing a drastic decrease in MTR values. These sites were not excluded from MTsat, because this metric is supposed to account for the T1 recovery effect^12^ as was indeed observed, with those sites now falling inside the 1σ interval. The site *tokyoSigna1* fell outside the 1σ interval because of issues related to image registration.

**Fig. 9:**
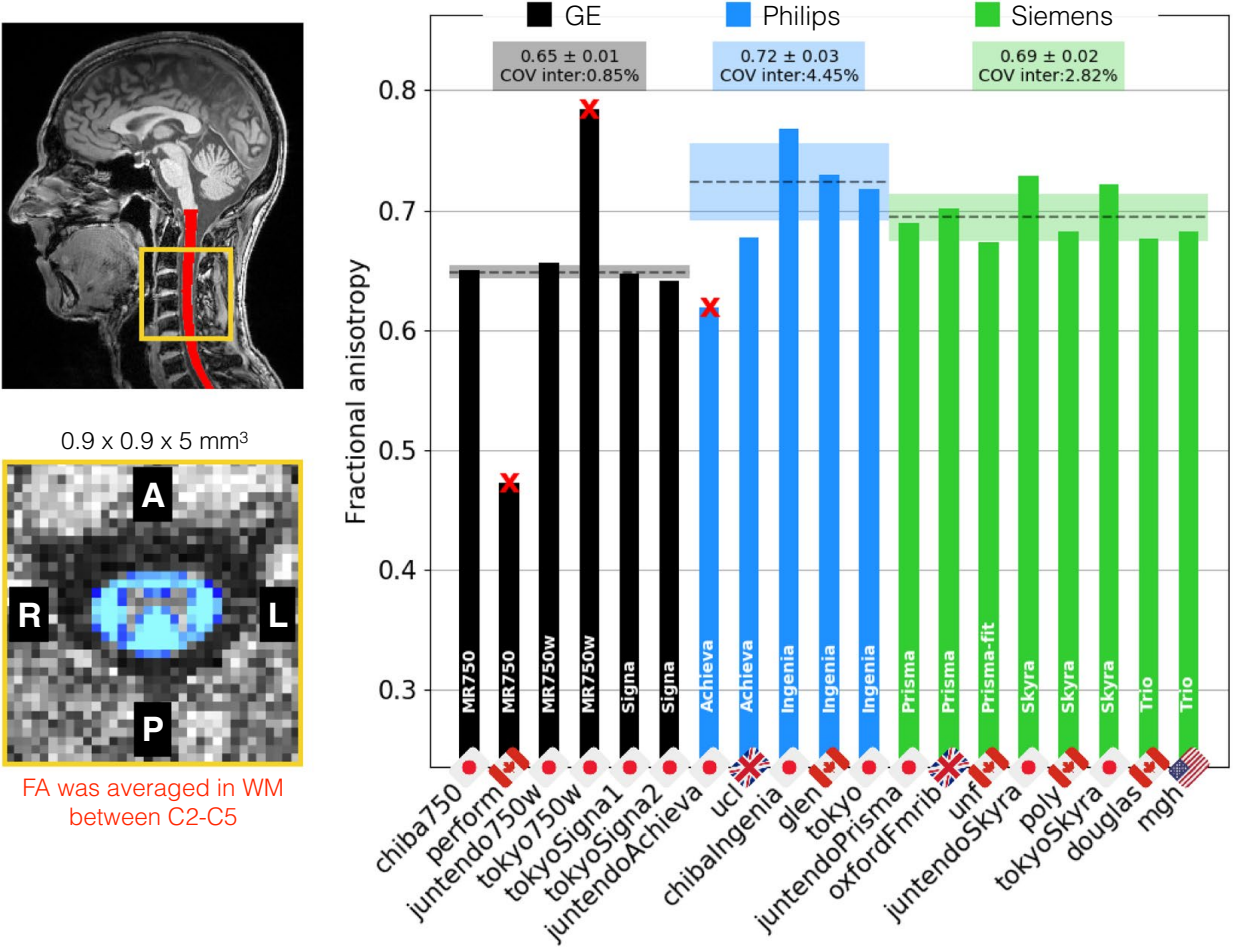
Results of the single subject study for the DWI protocol. The FA in the SC WM was averaged between the C2 and C5 vertebral levels. The following sites were excluded: *perform* (strong fat aliasing artifact), *tokyo750w* (poor shimming) and *juntendoAchieva* (no cardiac gating).

Figure 9 shows the average fractional anisotropy (FA) in WM across C2 and C5. The inter-site COVs were 0.8% for GE, 4.5% for Philips and 2.8% for Siemens. The inter-manufacturer difference was significant (p < 0.01), but the Tukey test showed no significant difference across pairwised manufacturers. One of the outliers (*tokyo750w*) was due to the absence of the FOCUS license, which led us to rely on saturation bands to prevent aliasing. However, those were not efficient (likely due to poor shimming in the region), with poor fat saturation efficiency that yielded spurious diffusion tensor fits (e.g. FA >1 or <0).

Average +/− standard deviation (SD) and COVs for mean diffusivity were, respectively, (0.62 +/− 0.03) mm^2^/s and 5.6% for GE, (1.00 +/− 0.06) mm^2^/s and 5.71% for Philips, and (1.01 +/− 0.05) mm^2^/s and 4.83% for Siemens. Average +/− SD and COVs for radial diffusivity were, respectively, (0.36 +/− 0.03) mm^2^/s and 7.21% for GE, (0.51 +/− 0.06) mm^2^/s and 11.34% for Philips, and (0.54 +/− 0.03) mm^2^/s and 6.37% for Siemens.

### Quantitative results: Multiple subjects

As in the case of the single subject data, all images were visually inspected to ensure that there were no significant errors in the masks used to average the signals in the SC, WM or GM and any errors were manually corrected. Complete metrics and statistical tests are available in the r20201130 release assets (https://github.com/spine-generic/data-multi-subject/releases/download/r20201130/results.zip). Interactive plots are available on the *spine generic* website (https://spine-generic.readthedocs.io/en/latest/analysis-pipeline.html#results).

**Fig. 10:**
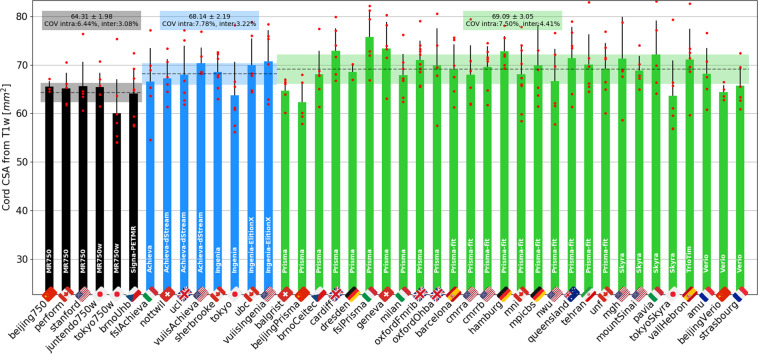
Results of multi-subject study for the T1w scan. As in the single subject study, the cross-sectional area of the SC was averaged between the C2 and C3 vertebral levels. Black, blue and green bars respectively correspond to GE, Philips and Siemens, with the manufacturer’s model indicated in white letters on each bar. The following participants were excluded from the statistics: *balgrist01* (motion), *beijingGE04* (motion), *mniS06* (motion), *mountSinai03* (participant repositioning), *oxfordFmrib04* (participant repositioning), *pavia04* (motion) and *perform06* (motion).

In Figure 10 we show the multi-subject, multi-center results for the SC CSA (averaged between C2 and C3) obtained from the T1w scan. The intra-site COVs were averaged for each manufacturer and found to be all just under 7.8%. The inter-site COVs (and inter-site ANOVA p-values) were 3.08% (p = 0.52) for GE, 3.22% (p = 0.44) for Philips and 4.41% (p = 0.12) for Siemens. The inter-manufacturer difference was significant (p = 0.0007), with the Tukey test showing significant differences between GE and Philips (p-adjusted < 0.01), and between GE and Siemens (p-adjusted < 0.01).

**Fig. 11:**
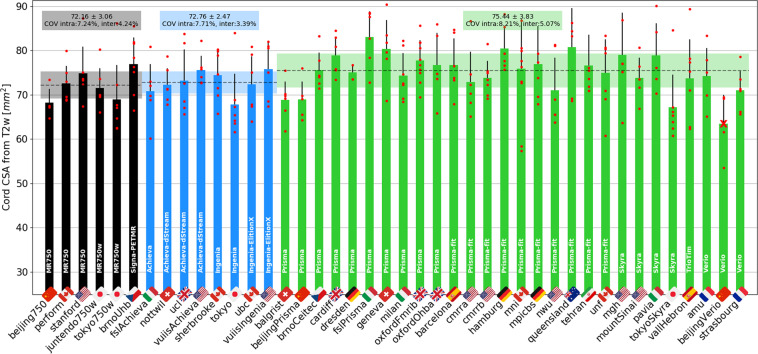
Results of multi-subject study for the T2w scan. The cross-sectional area of the SC was averaged between the C2 and C3 vertebral levels. The Siemens site *beijingVerio* was excluded from statistics (red cross) due to different TR and FA causing biases in the segmentation volume. The following participants were excluded: *oxfordFmrib04* (T1w scan was not aligned with other contrasts due to participant repositioning), *pavia04* (motion) and *mountSinai03* (participant repositioning).

Figure 11 has the CSAs obtained from the T2w scans (also averaged between C2 and C3). Again, intra-site COVs were close to 8%. Inter-site COVs (and ANOVA results) were 4.24% (p = 0.13) for GE, 3.39% (p = 0.35) for Philips, and 5.07% (p = 0.004) for Siemens. The inter-manufacturer difference was not significant (p = 0.17).

**Fig. 12:**
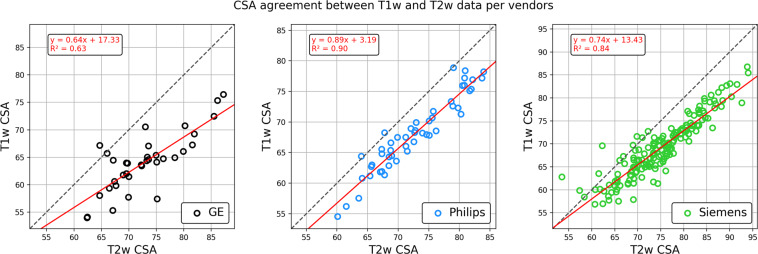
Relationship between CSA calculated from the T1w vs. T2w scans. The same site and participants excluded in Figs 10 and 11 were also excluded here.

Interestingly, T2w images were found to lead to larger cord CSAs than T1w images. In Figure 12 we show the relationship between T1w and T2w cord CSAs for all 3 manufacturers. Linear regressions led to *R*^2^ values that ranged from 0.63 for GE scanners (note that the same sequence was not used for all GE scanners) to 0.90 for Philips scanners.

**Fig. 13:**
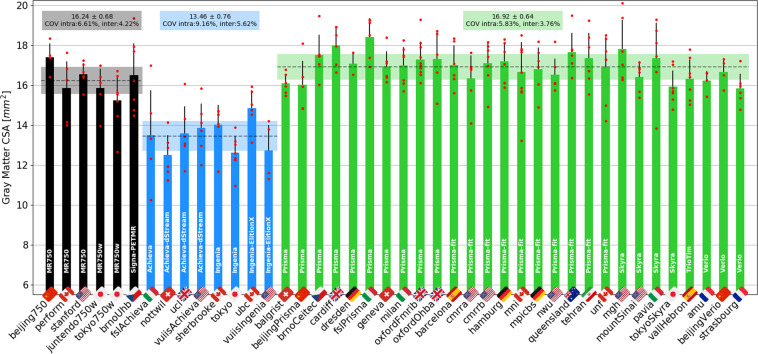
Gray matter CSA computed after automatic gray matter segmentation on the ME-GRE scan and averaged between C3 and C4 vertebral levels. The following participants were excluded due to motion artifacts: *amu03*, *fslAchieva04*, *vuiisIngenia04* and *vuiisIngenia05*.

Figure 13 shows the GM CSA, averaged across C3 and C4. The intra-site COV ranges from 5.83% (Siemens) to 9.16% (Philips). The inter-site COVs (and inter-site ANOVA p-values) were 4.22% (p = 0.14) for GE, 5.62% (p = 0.03) for Philips, and 3.76% (p = 0.005) for Siemens. The inter-manufacturer difference was significant (p = 2.3·10^−13^), with the Tukey test showing significant differences between GE and Philips (p-adjusted < 0.01), and between Philips and Siemens (p-adjusted < 0.01). The larger intra-site COV on Philips and the significantly lower values are likely due to the fact that some Philips sites used older versions of the consensus protocol, which produced lower contrast between white and gray matter and, as a result, less reliable gray matter segmentations.

**Fig. 14:**
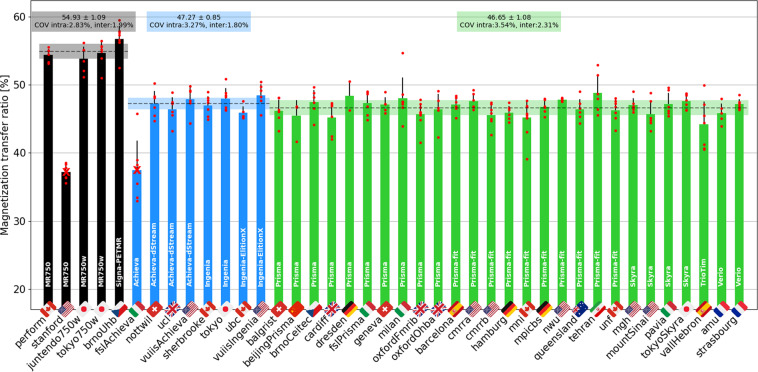
MTR results computed from the GRE-MT0 and GRE-MT1 scans and averaged in the SC WM between the C2 and C5 vertebral levels. The following sites were removed from the statistics: *stanford* (large difference in the TR), *fslAchieva* (wrong field of view (FOV) placement). The following participants were also removed: *beijingPrisma04* (different coil selection, shim value and FOV placement between MT1 and MT0), *geneva02* (FOV positioning changed between MT1 and MT0), *oxfordFmrib04* (T1w scan was not aligned with other contrasts due to participant repositioning).

Figure 14 shows MTR results averaged between C2 and C5. The intra-site COVs were averaged for each manufacturer and found to be all under 3.6%. The inter-site COVs (and inter-site ANOVA p-values) were 2.0% (p = 0.03) for GE, 1.8% (p = 0.17) for Philips, and 2.3% (p < 0.01) for Siemens. The inter-manufacturer difference was significant (p < 0.01), with the Tukey test showing significant differences between GE and Philips (p-adjusted = 0.02), and between GE and Siemens (p-adjusted = 0.01).

**Fig. 15:**
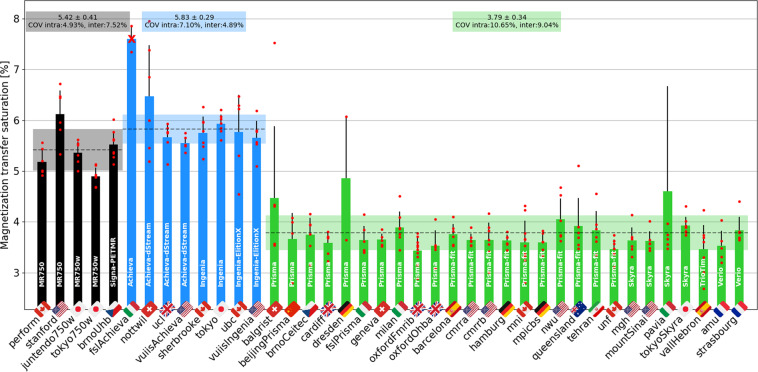
MTsat results computed from the GRE-MT0, GRE-MT1 and GRE-T1w scans and averaged in the SC WM between the C2 and C5 vertebral levels. The following site was removed from the statistics: *fslAchieva* (wrong FOV placement). The following participants were also removed: *beijingPrisma04* (different coil selection, shim value and FOV placement between MT1 and MT0), *geneva02* (FOV positioning changed between MT1 and MT0), *oxfordFmrib04* (T1w scan was not aligned with other contrasts due to participant repositioning).

Figure 15 shows MTsat results, also averaged between C2 and C5. The intra-site COVs were all under 11%. The inter-site COVs (and inter-site ANOVA p-values) were 7.5% (p < 0.01) for GE, 4.9% (p = 0.11) for Philips, and 9.0% (p = 0.09) for Siemens. The inter-manufacturer difference was significant (p < 0.01), with the Tukey test showing significant differences between GE and Philips (p-adjusted = 0.04), between GE and Siemens (p-adjusted <0.01), and between Philips and Siemens (p-adjusted < 0.01). Some outliers have notable impacts on the standard deviations: *nottwil03*, *nottwil04*, *pavia05*. These outliers are likely caused by poor image quality due to participant motion on the MT0 scan (see the full reports on the Github issue https://github.com/spine-generic/data-multi-subject/issues/36). Interestingly, these participants did not produce such outliers on the MTR results (which is computed from the MT1 and MT0 scans), and the T1w scan looked visually normal. We therefore decided to keep these participants on the figure in order to highlight possible implications about the reliability of the MTsat measures as a myelin biomarker (see discussion). We also decided to keep the *stanford* site (removed for MTR computation), because the T1 recovery effect induced by the different TR compared to other sites is supposed to be taken into account by the additional GRE-T1w scan when computing the MTsat metric, as is indeed confirmed in the figure (average MTsat for this site falls within the 1σ-2σ interval).

**Fig. 16:**
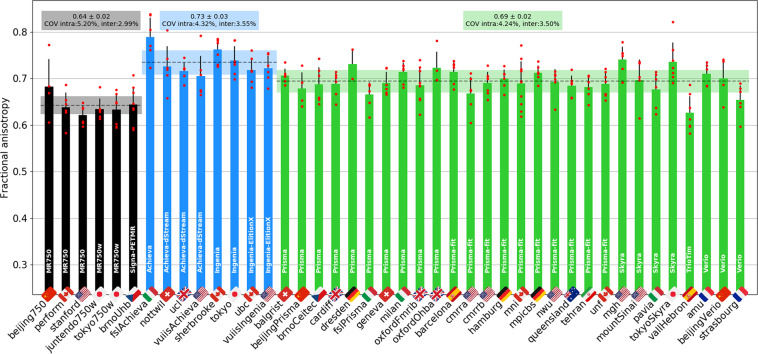
Results of multi-subject study for the DWI scan. The FA of the SC WM was averaged between the C2 and C5 vertebral levels. The following participants were excluded: *beijingPrisma03* (wrong FOV placement), *mountSinai03* (T2w was re-acquired, causing wrong T2w to DWI registration), *oxfordFmrib04* (participant repositioning) and *oxfordFmrib01* (registration issue).

Figure 16 shows FA results from the DWI scans, averaged between C2 and C5. The intra-site COVs were averaged for each manufacturer and found to be all under 5.2%. The inter-site COVs (and inter-site ANOVA p-values) were 3.0% (p = 0.25) for GE, 3.6% (p < 0.01) for Philips and 3.5% (p < 0.01) for Siemens. The inter-manufacturer difference was significant (p < 0.01), with the Tukey test showing significant differences between GE and Philips (p-adjusted < 0.01), between GE and Siemens (p-adjusted < 0.01), and between Philips and Siemens (p-adjusted < 0.01). Average +/− SD and inter-site COVs for mean diffusivity were, respectively, (0.73 +/− 0.09) mm^2^/s and 12.52% for GE, (0.97 +/− 0.08) mm^2^/s and 7.82% for Philips, and (0.99 +/− 0.04) mm^2^/s and 4.40% for Siemens. Average +/− SD and COVs for radial diffusivity were, respectively, (0.42 +/− 0.04) mm^2^/s and 10.31% for GE, (0.48 +/− 0.06) mm^2^/s and 12.25% for Philips, and (0.52 +/− 0.05) mm^2^/s and 8.91% for Siemens.

### Differences in qMRI results between manufacturers

Before discussing differences across and within manufacturers, we would like to stress that results presented here will become further refined with time because, as for any neuroimaging analysis pipeline, the algorithms evolve. Moreover, visual QC and manual corrections are prone to human error. We therefore encourage users of this living database to provide feedback. As it is an open source project, contributions are welcome. Also, as future participants are added, the statistics will be updated.

#### Spinal cord CSA

Within manufacturers, SC CSAs showed a maximum inter-site COV of 2.4% for the single subject study and 5% for the multi-subject study, for both T1w and T2w contrasts, which is highly encouraging. Overall, intra-site COVs were higher than inter-site COVs, which is expected because CSAs are known to vary substantially across individuals^20^. Hence, taking the mean within each site and comparing it across sites somewhat smooths this inherent inter-individual variability, putting aside geographical differences. This could be the goal of follow-up investigations.

Regardless of the manufacturer, intra-site COVs were about two-fold higher for SC CSAs (8%) compared to MTR and DTI-FA (4–5%). This result is not surprising, considering that, as noted above, SC size is known to vary across healthy adults, while white matter microstructure (which MTR and DTI-FA measure) is not expected to vary much between healthy individuals^21^. Note that there is no conclusive evidence of a correlation of SC CSA with age^22^, although some studies do report smaller cord area in older participants^23,24^. There is currently no accepted consensus on an effective and reliable normalization method for SC CSA^20^. Given that CSA is a widely used biomarker for neurodegenerative diseases such as MS, reducing that inter-subject variance is a much needed goal for the research community.

T1w scans showed slightly better intra- and inter-site COVs compared to T2w scans. This is rather surprising, given that T2w scans look visually cleaner, with a sharper SC/CSF border, and the fact that they are less prone to participant or SC motion artifacts. The SC CSAs obtained from the T1w scans were significantly lower for GE scanners, compared to both Philips and Siemens, whereas for T2w scans, the CSA was comparable across all three manufacturers. Variability of CSA across manufacturers could be due to (i) sequence parameters and/or reconstruction filters (e.g. smoothing) that alter the boundary definition, and/or (ii) differing field-strength between manufacturers (2.89 T for Siemens, 3.00 T for GE and Philips MRI) that change the apparent tissue contrasts^25^.

Interestingly, the SC CSA was overall higher on T2w vs. T1w sequences (see Fig. 12). The sensitivity of image contrast to CSA measurements has already been reported in a study comparing T2w SPACE and T1w MPRAGE sequences^26^, and in another study comparing T1w MPRAGE (3D-TFE) and 3D phase sensitive inversion recovery (PSIR) sequences^27^. As discussed elsewhere^28^, discrepancies in measurements across MR sequences and parameters could be caused by the slightly darker contour of the T2w image, accentuating partial volume effects with the surrounding CSF, T2* blurring, Gibbs ringing, motion and flow artifacts. These differences would thus change the identification of the SC boundaries by either the user (in case of manual segmentation) or an algorithm (in case of automated segmentation). It is worth noting that the type of MRI contrast can also impact the physical appearance of the boundaries. For example, the dura mater has a relatively short T2* value and hence its apparent location varies with the choice of TE in gradient echo sequences^29^. Age-related increases in iron deposition in the dura mater can also lead to CSA under-estimation, due to T2* reduction, which can be a confounding factor in longitudinal studies.

In order to measure CSA in retrospective or longitudinal studies, we therefore recommend sticking to exactly the same sequence and parameters. Users of our proposed protocols have the option of deriving the SC CSA from T1w or T2w images. While the two contrasts did lead to different SC CSA values, these have been modeled for each manufacturer. This means that when users compare SC CSA values that were obtained from different contrasts, they can account for differences between them by either acquiring sufficient data themselves, using our protocol and modeling the relationship between T1w and T2w SC CSA, or by using our estimated regression coefficients linking T1w CSA with T2w CSA (Fig. 12).

#### Gray matter CSA

In terms of the GM CSA, for the multi-subject dataset, GM CSAs showed a maximum inter-site COV of 5.6% (3.5% for the single subject dataset), which is highly encouraging, especially considering the small size of the GM, making CSA measures very sensitive to segmentation errors. Also worth mentioning is the inter-site standard deviation ranging from 0.64 to 0.76 mm^2^ (0.41 to 0.57 mm^2^ for single subject), which is remarkable considering that the effective in-plane spatial resolution of the image is 0.5 × 0.5 mm^2^, i.e., the precision is roughly the size of the pixel.

Philips scanners led to significantly lower CSA values here and also larger intra-site COVs, which is likely due to the fact that some Philips sites used older versions of the consensus protocol that produced lower contrast between white and gray matter and, as a result, less reliable gray matter segmentations. The current Philips protocol has different echo times and an increased saturation band power. The latter has the effect of generating a greater MT effect and, consequently, improved WM/GM contrast. The only site benefiting from these changes was *ubc*, which explains the GM CSA values being slightly closer here to those of the Siemens and GE sites.

#### Magnetization transfer

The MT protocol includes MTR and MTsat metrics, both of which are sensitive to myelin loss^30,31^. Owing to the use of GRE-T1w images (in addition to the MT1 and MT0 scans), MTsat is less sensitive to T1 recovery effects^12,32^, as has been confirmed by results from both the single- and multi-subject studies. However, this benefit is largely outweighed by it being noisier than MTR with maximum intra- and inter-site COVs of 11% and 9%, respectively, versus 4% and 2.3% for MTR. On the other hand, the higher COVs may be compensated by a higher sensitivity to myelin loss, given that myelin content appears to be more closely related to MTsat than to MTR^30^. This warrants further investigation in a patient population exhibiting abnormal myelination. Despite these somewhat discouraging results for the MTsat and T1 metrics, the GRE-T1w scan could still be kept in the *spine generic* protocol because it is short (~1 min) and could be useful for detecting hypointense lesions.

We otherwise noticed larger differences for the GE site compared to Philips and Siemens, which is likely attributed to the different MT pulse shape (Fermi for GE vs. sinc for Philips, and Gaussian for Siemens), and possibly different offset frequencies and energy. Another potential source of difference is that the acquisition matrix for the GE sites had to be reduced to 192 (instead of 256 for Philips/Siemens) because older software versions did not have ASSET (parallel imaging technique used by GE) on the GRE sequence that features the MT pulse.

#### Diffusion weighted imaging

As with MTR, DTI metrics showed very little intra- and inter-site variabilities. FA values were similar between Siemens and Philips, but significantly lower for GE. One possible explanation may lie in the different noise properties, which are known to impact DTI metrics^33^. Differences in noise properties could be related to receive coil properties, reconstruction of the images (GE data are reconstructed on a finer grid) or filters applied by the image reconstructor, among other factors. Another possible cause for the lower FA observed on the GE sites is the diffusion pulse sequence and the way diffusion gradients are played out (slew rate, mixing time, maximum gradient strength). For Siemens, the lower FA for the *vallHebron* (Tim Trio, within the 2σ-3σ interval) and *strasbourg* (Verio, within the 1σ-2σ interval) sites compared to other Siemens sites is likely caused by a much longer TE (99 ms for Trio and 95 ms for Verio, versus 55–60 ms for Skyra and Prisma), increasing noise amplitude with an impact in the DTI metrics. That said, *amu* and *beijingVerio* sites were also Verio, but their FA values were within the 1σ interval. Other DTI metrics followed the same trends in terms of intra- and inter-manufacturer variability, although COVs were higher, which could be explained by the less forgiving behaviour of these DTI metrics with respect to image quality (motion, ghosting, low signal-to-noise ratio).

Another factor which likely impacted the variability was the non-use/misuse of cardiac gating. As observed in the single subject study, DTI metrics were abnormal for sites that did not use cardiac gating, as this led to a sudden drop in signal not related to microscopic water diffusion (see Fig. 4g). The present study reiterates the benefits of cardiac gating in SC DWI experiments.

## Usage Notes

### BIDS convention

We recommend that researchers planning to contribute to the *spine generic* database or creating other databases check the validity of the json sidecars associated with BIDS datasets. This will help assess how well protocols are followed by different centers. For json files to contain the relevant information, it is necessary that (i) DICOM fields include the relevant fields themselves, including the obvious (TR, TE, flip angle) as well as lesser known parameters that can have a strong impact on the computed metrics (water excitation, fat saturation, monopolar vs. bipolar readout, etc.), and (ii) that these fields are populated in the json files. Checking these parameters as well as the files and folder names can be automatized via continuous integration (e.g., GitHub Actions, as used in the present study).

Another advantage of the BIDS convention is that it enables the standardization of the inputs/outputs of complex analysis pipelines, or so-called “BIDS Apps” (https://bids-apps.neuroimaging.io/). For example, the proposed analysis pipeline for the *spine generic* project can be applied ‘as is’ to another dataset organized according to BIDS.

### Concluding remarks and future directions

To the best of our knowledge, this study features the first “large-scale” multi-center SC qMRI datasets ever acquired and made public. These datasets are shared according to the ‘Findable, Accessible, Interoperable and Reusable’ (FAIR) principles^34^. The normative values from the multi-subject dataset could serve as age-matched healthy control references. More generally, these datasets will be useful for developing new image processing tools dedicated to the SC, and the fact that they are public and version-tracked with git-annex technology makes it possible for researchers to compare tools with the same data.

Lastly, important efforts were deployed to make the data analysis methods fully transparent and the results reproducible. The analysis is fully automated - aside from minor manual corrections when necessary-, minimizing user bias and facilitating large multi-center studies. We hope this analysis framework can serve as an example for future studies and we encourage researchers to use it. The SC MRI community has initiated a forum (https://forum.spinalcordmri.org/) to encourage discussions about these open-access datasets, and to pitch new ideas for subsequent analyses and acquisitions.

In a time where reproducibility of scientific results is a major concern^35^, we believe a consensus acquisition protocol along with publicly-shared datasets and a transparent analysis pipeline provide a solid foundation for the field of SC qMRI so that, in the future, inclusion of the SC in neuroimaging protocols will become a “no-brainer”.

## Supplementary information

Supplementary Information

## Data Availability

Data were processed using Python and shell scripts contained in the spine-generic package (https://github.com/spine-generic/spine-generic/releases/tag/v2.6), which is distributed under the MIT license. A comprehensive procedure is described in the “Analysis pipeline” section of the spine generic website (https://spine-generic.rtfd.io/). This procedure includes the list of dependent software packages to install, a step-by-step analysis procedure with a list of commands to run, a procedure for quality control and for manual correction of intermediate outputs (e.g. cord segmentation and vertebral labeling). The procedure includes embedded video tutorials and has been tested by external users. The analysis documentation also includes a section on how to generate the static figures that are shown in this article (in PNG format) as well as the interactive figures embedded in the spine-generic website. Notable software used in this study include: the Spinal Cord Toolbox v5.0.1 (https://spinalcordtoolbox.com) to analyse the MRI data, pandas^36^ to perform statistics, plotly v4.12.0 (https://plotly.com) to display the interactive plots, brainsprite v0.13.3 (https://brainsprite.github.io/) for embedding in the online documentation an interactive visualization of example datasets, pybids^37^ for checking the acquisition parameters on the BIDS datasets, FSLeyes v0.34.0 (https://fsl.fmrib.ox.ac.uk/fsl/fslwiki/FSLeyes) for manually-correcting the segmentations.

## Online-only Table

**Online-only Table 1:** List of centers which contributed to the “Spinal Cord MRI Public Database (Multi-subject)”.

Acronym	Center	Contact	Brand, model	Software
beijing750	Beijing Tiantan Hospital, Capital Medical University	Y. Kong	GE 750	DV24.0_R01_1344.a
		Y. Liu		
		Y. Duan		
perform	PERFORM Center, Concordia University	J. Cohen-Adad	GE 750	DV25.1
		H. Benali		
stanford	Richard M. Lucas Center, Stanford University School of Medicine, Stanford, CA, USA	C. Law	GE 750	DV26.0_R01
		K.A. Weber II		
juntendo750w	Juntendo University School of Medicine, Tokyo, Japan	A. Hagiwara	GE, Discovery 750w	DV26.0_R02
tokyo750w	The University of Tokyo, Tokyo, Japan	K. Kamiya	GE, 750w	DV24.0
		Y. Suzuki		
brnoUhb	The University Hospital Brno, Department of Radiology and Nuclear Medicine, Czech Republic	M. Dostál	GE, Signa PETMR	MP24
		M. Keřkovský		
fslAchieva	Santa Lucia Foundation IRCCS, Rome, Italy	M. Fratini	Philips, Achieva	5.4
		F. Giove		
fslPrisma	Santa Lucia Foundation IRCCS, Rome, Italy	M. Fratini	Siemens,	VE11E
		F. Giove	Prisma	
nottwill	Swiss Paraplegic Centre, Nottwil, Switzerland	P. Wyss	Philips, Achieva	R5.4
		M. Abramovic		
		C. Arneitz		
sherbrooke	Centre de Recherche CHUS, CIMS, Sherbrooke, Canada	M. Descoteaux	Philips, Ingenia	R5.3
tokyo	The University of Tokyo, Tokyo, Japan	K. Kamiya	Philips, Ingenia	R5.3
		Y. Suzuki		
ucl	Queen Square MS Centre, University College London (UCL), London, UK	C. Gandini Wheeler-Kingshott	Philips, Ingenia CX	R5.4
		F. Grussu		
		R.S. Samson		
		M. Battiston		
		M.C. Yiannakas		
ubc	University of British Columbia MRI Research Centre, Vancouver Canada	L. Barlow	Philips, Ingenia-ElitionX	R5.5
		A.V. Dvorak		
		S. Kolind		
		C. Laule		
vuiisAchieva	Vanderbilt University Institute of Imaging Science	K.P. O’Grady	Philips, Achieva-dStream	R5.3
		A.J.E. Combes		
		S.A. Smith		
vuiisIngenia	Vanderbilt University Institute of Imaging Science	S. Smith	Philips, Ingenia-ElitionX	R5.5
		B. Landman		
		K. O’Grady		
		A. Combes		
balgrist	Spinal Cord Injury Center University Balgrist	M. Seif	Siemens, Prisma	VE11C
		P. Freund		
beijingPrisma	Beijing Tiantan Hospital, Capital Medical University	Y. Kong	Siemens, Prisma	VE11C
		Y. Liu		
		Y. Duan		
brnoCeitec	CEITEC Brno and University Hospital Olomouc, Czech Republic	T. Horák	Siemens, Prisma	VE11C
		P. Kudlička		
		J. Valošek		
cardiff	Cardiff University, Wales, UK	G. Tackley	Siemens, Prisma	VE11C
		S. Kusmia		
		R. Wise		
dresden	Institut für Diagnostische und Interventionelle Neuroradiologie, Technische Universität Dresden, Germany	P. Kuntke	Siemens, Prisma	VE11C
		H.H. Kitzler		
		T. Wesemann		
geneva	Campus Biotech, Genève	N. Kinany	Siemens, Prisma	VE11C
		L. Mattera		
		D. Van De Ville		
milan	IRCCS Fondazione Don Carlo Gnocchi, Milan, Italy	M.M. Laganà	Siemens, Prisma	VE11C
oxfordFmrib	Wellcome Centre For Integrative Neuroimaging, FMRIB, Nuffield Department of Clinical Neurosciences, University of Oxford, Oxford	D. Papp	Siemens, Prisma	VE11C
		A. Smith		
oxfordOhba	Oxford Centre for Human Brain Activity, Wellcome Centre for Integrative Neuroimaging, Department of Psychiatry, University of Oxford	D. Papp	Siemens, Prisma	VE11C
		A. Smith		
queensland	The University of Queensland, Centre for Advanced Imaging, Brisbane, QLD, Australia	N.D. Kurniawan	Siemens, Prisma-fit	VE11C
		M.J. Ruitenberg		
		M. Barth		
		N. Atcheson		
barcelona	Center of Neuroimmunology, Laboratory of Advanced Imaging in Neuroimmunological Diseases, Barcelona, Spain	E. Martinez-Heras	Siemens, Prisma-fit	VE11C
cmrra	Center for Magnetic Resonance Research, University of Minnesota, Minneapolis, MN, USA	P.-G. Henry	Siemens, Prisma-fit	VE11C
		J. Joers		
		R. Labounek		
		C. Lenglet		
		I. Nestrasil		
cmrrb	Center for Magnetic Resonance Research, University of Minnesota, Minneapolis, MN, USA	P.-G. Henry	Siemens, Prisma-fit	VE11C
		J. Joers		
		R. Labounek		
		C. Lenglet		
		I. Nestrasil		
hamburg	Department of Systems Neuroscience, University Medical Center Hamburg-Eppendorf, Hamburg, Germany	A. Tinnermann	Siemens, Prisma-fit	VE11C
		J. Finsterbusch		
mni	McConnell Brain Imaging Centre, Montreal Neurological Institute	B. De Leener	Siemens, Prisma-fit	VE11C
		J. Doyon		
mpicbs	Max Planck Institute for Human Cognitive and Brain Sciences, Leizpig, Germany	F. Eippert	Siemens, Prisma-fit	VE11C
		T. Leutritz		
		N. Weiskopf		
nwu	Northwestern University School of Medicine, Chicago, IL, USA	H. Karbasforoushan	Siemens, Prisma-fit	VE11C
		T. Parrish		
		Z. Smith		
tehran	National Brain Mapping Lab, Tehran, Iran	A. Khatibi	Siemens, Prisma-fit	VE11B
unf	Functional Neuroimaging Unit, IUGM, Montreal, Canada	J. Cohen-Adad	Siemens, Prisma-fit	VE11C
		A. Foias		
mgh	Athinoula A. Martinos Center for Biomedical Imaging, Charlestown, MA, USA	R.L. Barry	Siemens, Skyra	VE11C
pavia	IRCCS Mondino Foundation, Pavia, Italy	G. Savini	Siemens, Skyra	VD13C
tokyoSkyra	The University of Tokyo, Tokyo, Japan	K. Kamiya	Siemens, Skyra	VE11C
		Y. Suzuki		
mountSinai	BMEII, Icahn School of Medicine at Mount Sinai, New York, NY USA	J. Xu	Siemens, Skyra	VE11C
		A. Seifert		
		J-W. Kim		
vallHebron	Neuroradiology Section, Vall Hebron University Hospital. Barcelona, Spain	D. Pareto	Siemens, Trio Tim	VB17
		À. Rovira		
amu	Centre de Résonance Magnétique Biologique et Médicale (CRMBM-CEMEREM), Hôpital de la Timone, AP-HM, CNRS, Aix-Marseille Université, Marseille, France	V. Callot	Siemens, Verio	VB17
beijingVerio	Beijing Tiantan Hospital, Capital Medical University	Y. Kong	Siemens, Verio	VB22
		Y. Liu		
		Y. Duan		
strasbourg	Université de Strasbourg, CNRS, ICube, FMTS, Strasbourg, France	P. De Sousa	Siemens, Verio	VB17
