# Author Correction: Open-access quantitative MRI data of the spinal cord and reproducibility across participants, sites and manufacturers

**DOI:** 10.1038/s41597-021-01044-0

**Published:** 2021-09-23

**Authors:** Julien Cohen-Adad, Eva Alonso-Ortiz, Mihael Abramovic, Carina Arneitz, Nicole Atcheson, Laura Barlow, Robert L. Barry, Markus Barth, Marco Battiston, Christian Büchel, Matthew Budde, Virginie Callot, Anna J. E. Combes, Benjamin De Leener, Maxime Descoteaux, Paulo Loureiro de Sousa, Marek Dostál, Julien Doyon, Adam Dvorak, Falk Eippert, Karla R. Epperson, Kevin S. Epperson, Patrick Freund, Jürgen Finsterbusch, Alexandru Foias, Michela Fratini, Issei Fukunaga, Claudia A. M. Gandini Wheeler-Kingshott, Giancarlo Germani, Guillaume Gilbert, Federico Giove, Charley Gros, Francesco Grussu, Akifumi Hagiwara, Pierre-Gilles Henry, Tomáš Horák, Masaaki Hori, James Joers, Kouhei Kamiya, Haleh Karbasforoushan, Miloš Keřkovský, Ali Khatibi, Joo-Won Kim, Nawal Kinany, Hagen H. Kitzler, Shannon Kolind, Yazhuo Kong, Petr Kudlička, Paul Kuntke, Nyoman D. Kurniawan, Slawomir Kusmia, René Labounek, Maria Marcella Laganà, Cornelia Laule, Christine S. Law, Christophe Lenglet, Tobias Leutritz, Yaou Liu, Sara Llufriu, Sean Mackey, Eloy Martinez-Heras, Loan Mattera, Igor Nestrasil, Kristin P. O’Grady, Nico Papinutto, Daniel Papp, Deborah Pareto, Todd B. Parrish, Anna Pichiecchio, Ferran Prados, Àlex Rovira, Marc J. Ruitenberg, Rebecca S. Samson, Giovanni Savini, Maryam Seif, Alan C. Seifert, Alex K. Smith, Seth A. Smith, Zachary A. Smith, Elisabeth Solana, Y. Suzuki, George Tackley, Alexandra Tinnermann, Jan Valošek, Dimitri Van De Ville, Marios C. Yiannakas, Kenneth A. Weber II, Nikolaus Weiskopf, Richard G. Wise, Patrik O. Wyss, Junqian Xu

**Affiliations:** 1 NeuroPoly Lab, Institute of Biomedical Engineering, Polytechnique Montreal, Montreal, QC Canada; 2 Functional Neuroimaging Unit, CRIUGM, Université de Montréal, Montreal, QC Canada; 3 Mila - Quebec AI Institute, Montreal, QC Canada; 4 Department of Radiology, Swiss Paraplegic Centre, Nottwil, Switzerland; 5 Centre for Advanced Imaging, The University of Queensland, Brisbane, Australia; 6 Department of Radiology, University of British Columbia, Vancouver, BC Canada; 7 Athinoula A. Martinos Center for Biomedical Imaging, Department of Radiology, Massachusetts General Hospital, Charlestown, MA USA; 8 Department of Radiology, Harvard Medical School, Boston, MA USA; 9 Harvard–Massachusetts Institute of Technology Health Sciences & Technology, Cambridge, MA USA; 10 School of Information Technology and Electrical Engineering, The University of Queensland, Brisbane, Australia; 11 NMR Research Unit, Queen Square MS Centre, UCL Queen Square Institute of Neurology, Faculty of Brain Sciences, University College London, London, UK; 12 Department of Systems Neuroscience, University Medical Center Hamburg-Eppendorf, Hamburg, Germany; 13 Department of Neurosurgery, Medical College of Wisconsin, Milwaukee, WI USA; 14 Aix-Marseille Univ, CNRS, CRMBM, Marseille, France; 15 APHM, Hopital Universitaire Timone, CEMEREM, Marseille, France; 16 Vanderbilt University Institute of Imaging Science, Vanderbilt University Medical Center, Nashville, TN USA; 17 Department of Computer and Software Engineering, Polytechnique Montreal, Montreal, Canada; 18 CHU Sainte-Justine Research Centre, Montreal, QC Canada; 19 Centre de Recherche CHUS, CIMS, Sherbrooke, Canada; 20 Sherbrooke Connectivity Imaging Lab (SCIL), Computer Science department, Université de Sherbrooke, Sherbrooke, Canada; 21 Université de Strasbourg, CNRS, ICube, Strasbourg, France; 22 UHB - University Hospital Brno and Masaryk University, Department of Radiology and Nuclear Medicine, Brno, Czech Republic; 23 McConnell Brain Imaging Centre, Montreal Neurological Institute, McGill University, Montreal, QC Canada; 24 Department of Physics and Astronomy, University of British Columbia, Vancouver, BC Canada; 25 Max Planck Institute for Human Cognitive and Brain Sciences, Leipzig, Germany; 26 Richard M. Lucas Center, Stanford University School of Medicine, Stanford, CA USA; 27 Spinal Cord Injury Center Balgrist, University of Zurich, Zurich, Switzerland; 28 Institute of Nanotechnology, CNR, Rome, Italy; 29 IRCCS Santa Lucia Foundation, Rome, Italy; 30 Department of Radiology, Juntendo University School of Medicine, Tokyo, Japan; 31 Department of Brain and Behavioural Sciences, University of Pavia, Pavia, Italy; 32 Brain MRI 3T Research Centre, IRCCS Mondino Foundation, Pavia, Italy; 33 MR Clinical Science, Philips Healthcare, Markham, ON Canada; 34 CREF - Museo storico della fisica e Centro studi e ricerche Enrico Fermi, Rome, Italy; 35 Radiomics Group, Vall d’Hebron Institute of Oncology, Vall d’Hebron Barcelona Hospital Campus, Barcelona, Spain; 36 Center for Magnetic Resonance Research, Department of Radiology, University of Minnesota, Minneapolis, MN USA; 37 Multimodal and functional imaging laboratory, Central European Institute of Technology (CEITEC), Brno, Czech Republic; 38 Department of Radiology, Toho University Omori Medical Center, Tokyo, Japan; 39 Department of Radiology, the University of Tokyo, Tokyo, Japan; 40 Interdepartmental Neuroscience Program, Feinberg School of Medicine, Northwestern University, Chicago, IL USA; 41 Department of Psychiatry and Behavioral Sciences, School of Medicine, Stanford University, Stanford, CA USA; 42 Centre of Precision Rehabilitation for Spinal Pain (CPR Spine), School of Sport, Exercise and Rehabilitation Sciences, College of Life and Environmental Sciences, University of Birmingham, Edgbaston, Birmingham, UK; 43 BioMedical Engineering and Imaging Institute (BMEII), Department of Radiology, Icahn School of Medicine at Mount Sinai, New York, NY USA; 44 Institute of Bioengineering/Center for Neuroprosthetics, Ecole Polytechnique Fédérale de Lausanne, Geneva, Switzerland; 45 Department of Radiology and Medical Informatics, University of Geneva, Geneva, Switzerland; 46 Institute of Diagnostic and Interventional Neuroradiology, Carl Gustav Carus University Hospital, Technische Universität Dresden, Dresden, Germany; 47 Department Of Medicine (Neurology), University of British Columbia, Vancouver, BC Canada; 48 CAS Key Laboratory of Behavioral Science, Institute of Psychology, Chinese Academy of Sciences, Beijing, China; 49 Department of Psychology, University of Chinese Academy of Sciences, Beijing, China; 50 Wellcome Centre For Integrative Neuroimaging, FMRIB, Nuffield Department of Clinical Neurosciences, University of Oxford, Oxford, UK; 51 CUBRIC, Cardiff University, Wales, UK; 52 Centre for Medical Image Computing (CMIC), Medical Physics and Biomedical Engineering Department, University College London, London, UK; 53 Epilepsy Society MRI Unit, Chalfont St Peter, UK; 54 Division of Clinical Behavioral Neuroscience, Department of Pediatrics, University of Minnesota, Minneapolis, MN USA; 55 Departments of Neurology and Biomedical Engineering, University Hospital Olomouc, Olomouc, Czech Republic; 56 IRCCS Fondazione Don Carlo Gnocchi ONLUS, Milan, Italy; 57 Departments of Radiology, Pathology & Laboratory Medicine, Physics & Astronomy; International Collaboration on Repair Discoveries (ICORD), University of British Columbia, Vancouver, BC Canada; 58 Division of Pain Medicine, Department of Anesthesiology, Perioperative and Pain Medicine, Stanford University School of Medicine, Stanford, CA USA; 59 Department of Neurophysics, Max Planck Institute for Human Cognitive and Brain Sciences, Leipzig, Germany; 60 Department of Radiology, Beijing Tiantan Hospital, Capital Medical University, Beijing, China; 61 Tiantan Image Research Center, China National Clinical Research Center for Neurological Diseases, Beijing, China; 62 Center of Neuroimmunology, Laboratory of Advanced Imaging in Neuroimmunological Diseases, Hospital Clinic Barcelona, Institut d’Investigacions Biomèdiques August Pi i Sunyer (IDIBAPS) and Universitat de Barcelona, Barcelona, Spain; 63 Fondation Campus Biotech Genève, 1202 Geneva, Switzerland; 64 Department of Radiology, Vanderbilt University Medical Center, Nashville, TN USA; 65 UCSF Weill Institute for Neurosciences, Department of Neurology, University of California San Francisco, San Francisco, CA USA; 66 Neuroradiology Section, Vall d’Hebron University Hospital, Barcelona, Spain; 67 E-health Centre, Universitat Oberta de Catalunya, Barcelona, Spain; 68 School of Biomedical Sciences, Faculty of Medicine, The University of Queensland, Brisbane, Australia; 69 University of Oklahoma Health Sciences Center, Oklahoma City, OK USA; 70 Department of Neurology, Faculty of Medicine and Dentistry, Palacký University and University Hospital Olomouc, Olomouc, Czech Republic; 71 Felix Bloch Institute for Solid State Physics, Faculty of Physics and Earth Sciences, Leipzig University, Leipzig, Germany; 72 Institute for Advanced Biomedical Technologies, Department of Neuroscience, Imaging and Clinical Sciences, “G. D’Annunzio University” of Chieti-Pescara, Chieti-Pescara, Italy

**Keywords:** Imaging techniques, Spinal cord diseases, Databases, Imaging techniques, Spinal cord diseases, Databases

Correction to: *Scientific Data* 10.1038/s41597-021-00941-8, published online 16 August 2021

In the original version of this Data Descriptor, the Figure 4 legend was a duplicate of the Figure 3 legend. The Figure 4 legend has now been corrected to read as follows in both the PDF and HTML versions of the Data Descriptor:

Examples of image artifacts: (a) T1w MPRAGE taken in the same participant (from the single subject database) at two different sites on a Siemens Prisma: oxfordFmrib (left) and juntendoPrisma (right). The slightly larger cervical lordosis on the left likely induced more pronounced cerebrospinal fluid (CSF) flow and SC motion resulting in the artifact shown in the axial view.  (b) T2w scans showing signal drops in the CSF likely due to a poorly-recovered CSF signal combined with flow effects. These two participants (beijingVerio01 and strasbourg03), were acquired with a flip angle of 180° instead of the recommended 120°, which likely explained the presence of those artifacts (longer TR was required for sufficient T1 recovery). (c) Axial view of ME-GRE scans with (fslAchieva04, 1st row) and without motion (brnoCeitec01, 2nd row), and axial view of GRE-MT0 with (fslAchieva04, 3rd row) and without motion (barcelona04, 4th row). (d) Mean DWI scan from a Philips site (ubc02, left panel) with a concatenated acquisition wherein odd slices are acquired during the first half of the entire acquisition (spanning all b-vectors) and the even slices are acquired during the second half. In the event of participant motion between those two acquisition sub-sets, apparent motion will be visible between the odd and even slices. When odd and even slices are acquired closer in time (in ascending/descending mode, or interleaved but sequentially within the same b-vector), this artifact is not visible (mountSinai03, right panel). Such an artifact could be problematic for image registration with regularization along the S-I axis, or for performing diffusion tractography. (e) b = 0 image from a DWI scan (perform02) acquired with poor shimming and resulting signal dropout. (f) Another example of poor shimming resulting in sub-efficient fat saturation, with the fat being aliased on top of the SC. Here we show the mean DWI scan of a participant from the single subject database (perform). (g) Effect of pulsatile movement on a non-cardiac gated acquisition (single subject, juntendoAchieva). Diffusion-weighted scans (sagittal view) acquired at three b-vecs fairly orthogonal to the SC (i.e., diffusion-specific signal attenuation should be minimum in the SC), showing abrupt signal drop at a few slices (red arrows), likely due to cardiac-related pulsatile effects.

