# Supplementary material for: Open-access quantitative MRI data of the spinal cord and reproducibility across participants, sites and manufacturers
Source: Sci Data. 2021 Aug 16;8:219. doi: 10.1038/s41597-021-00941-8 (PMC8368310; doi:10.1038/s41597-021-00941-8)
Supplement: Supplementary file 1 — Supplementary Information [file 41597_2021_941_MOESM1_ESM.pdf]

Supplementary material

Example T1w images 2

Example T2w images 3

Example multiecho GRE images 4

Example GRE T1w images 5

Example DWI data 6

Example T1w images

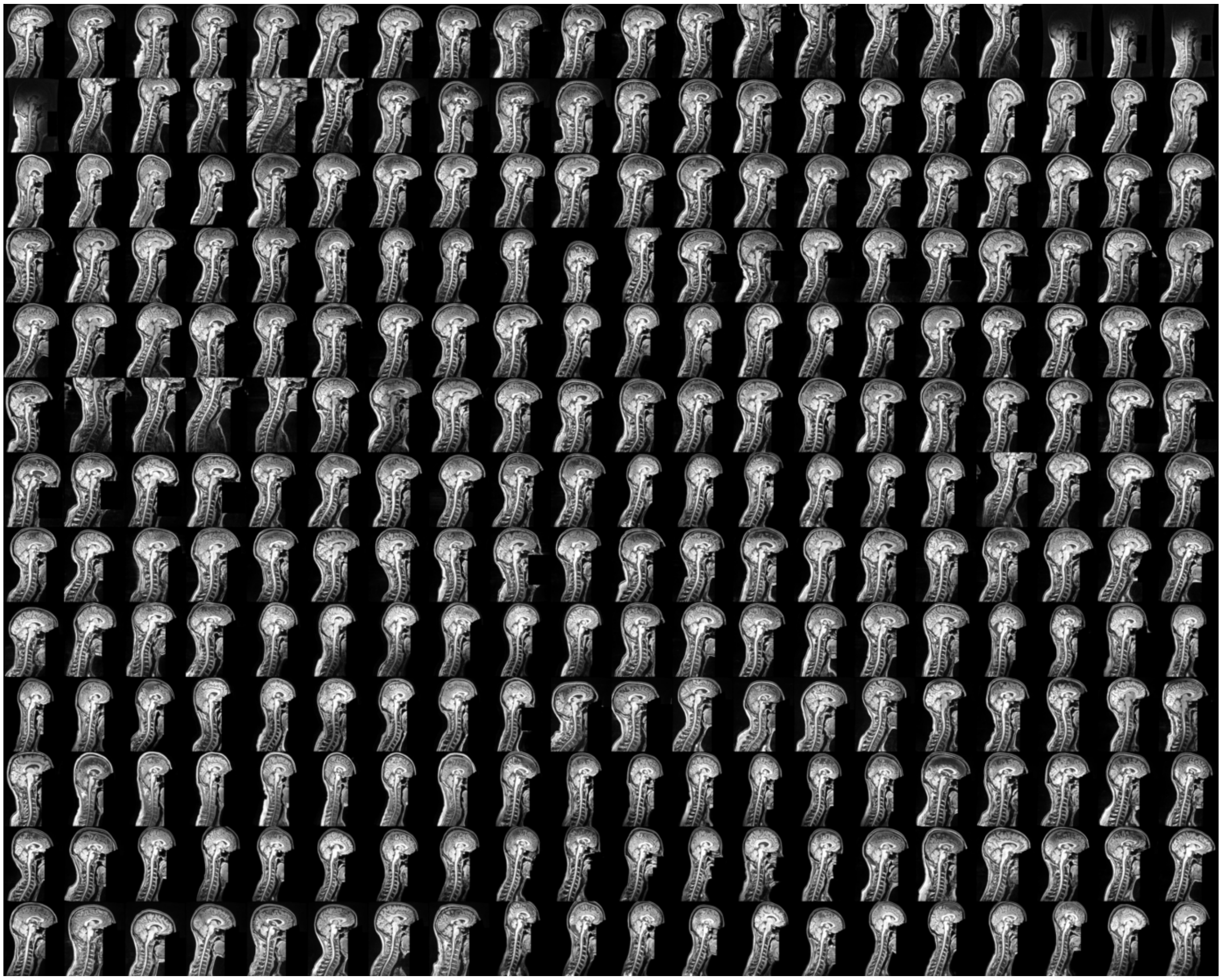

Figure S1. *T1w sagittal images of individual participants from the multi-subject database. Images were non-linearly aligned (slicewise translation along the right-left direction) using the spinal cord centerline obtained from the segmentation, in order to have the spinal cord aligned in the medial plane for better visual assessment. Enhance Local Contrast (CLAHE) filter was applied, and the signal range of every image was normalized across all participants to account for different signal quantization across vendors.*

Example T2w images

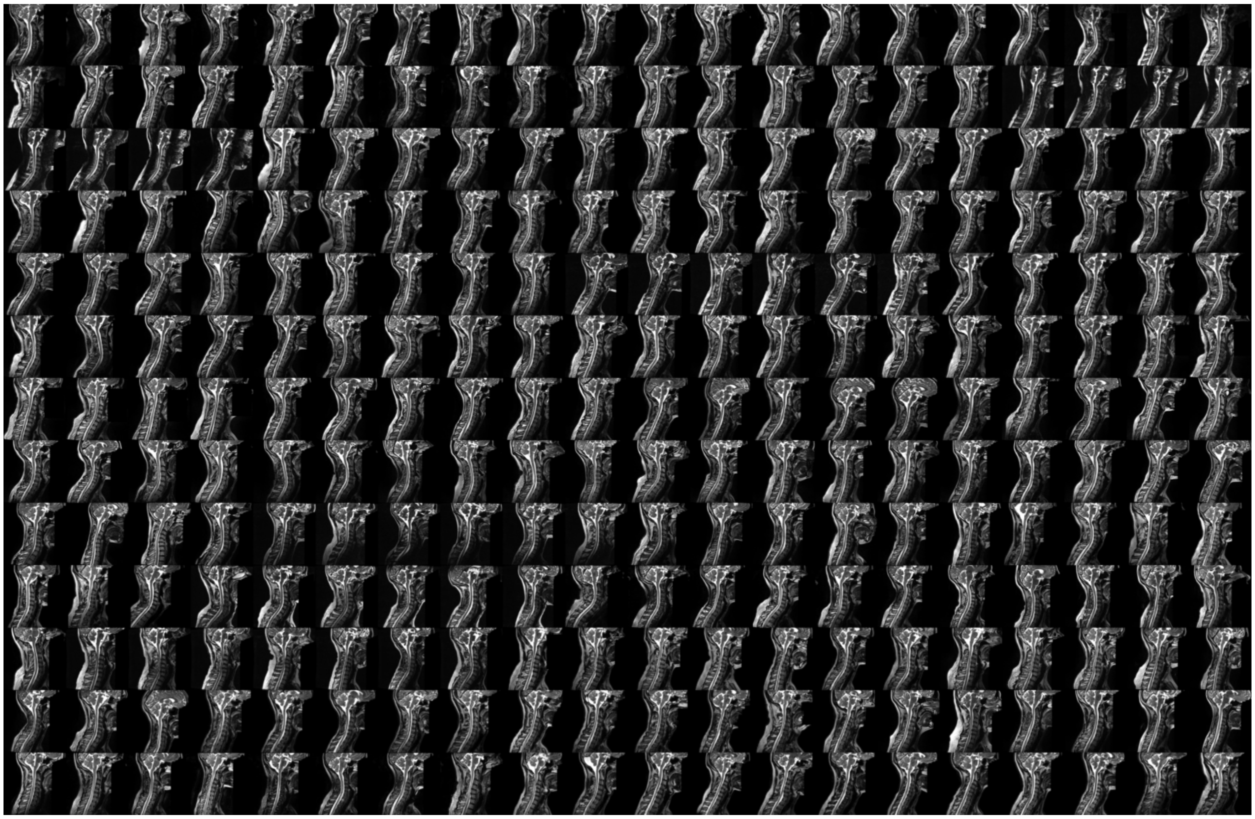

Figure S2. *T2w sagittal images of individual participants from the multi-subject database. Images were processed as in* ***Figure S1****.*

Example multiecho GRE images

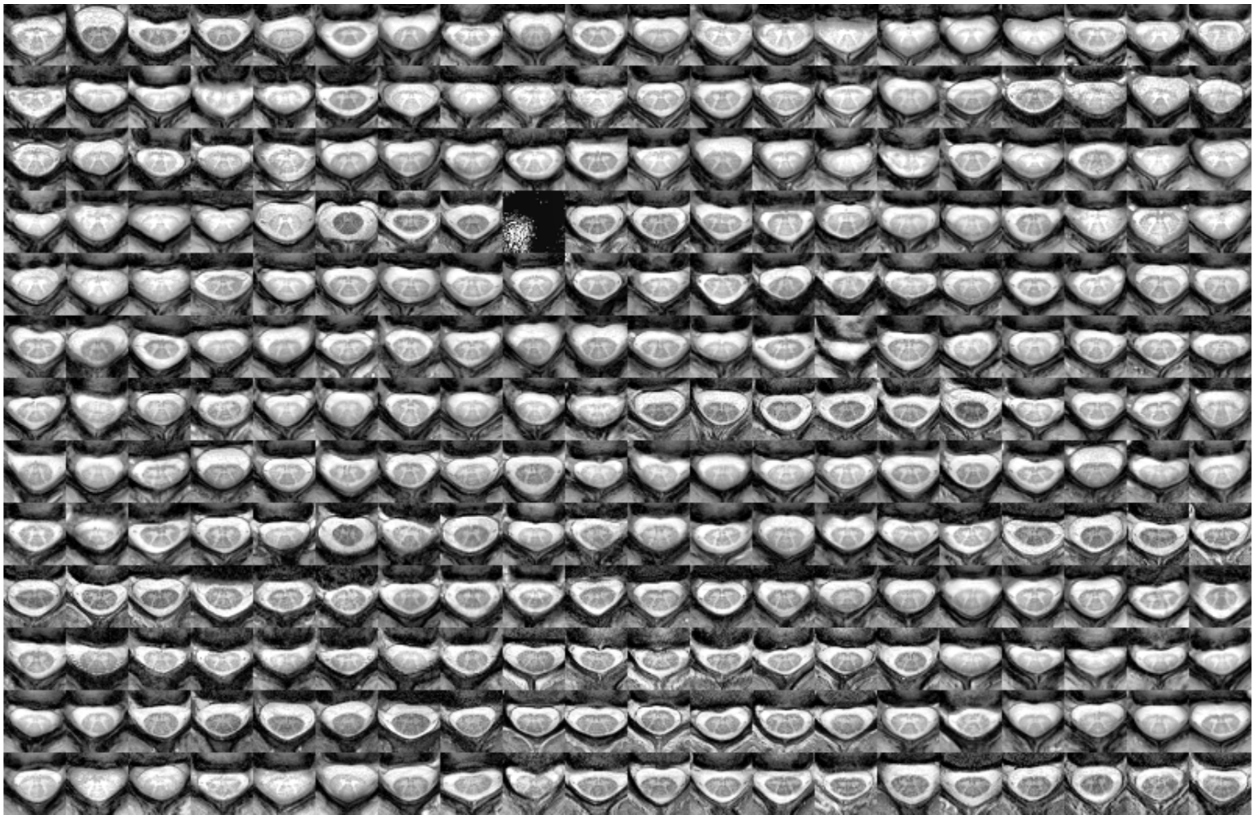

Figure S3. *T2*w axial images (mid-slice) of individual participants from the multi-subject database. The slice missing (row 4, column 9) is due to a mis-registration caused by the repositioning of a participant.*

Example GRE T1w images

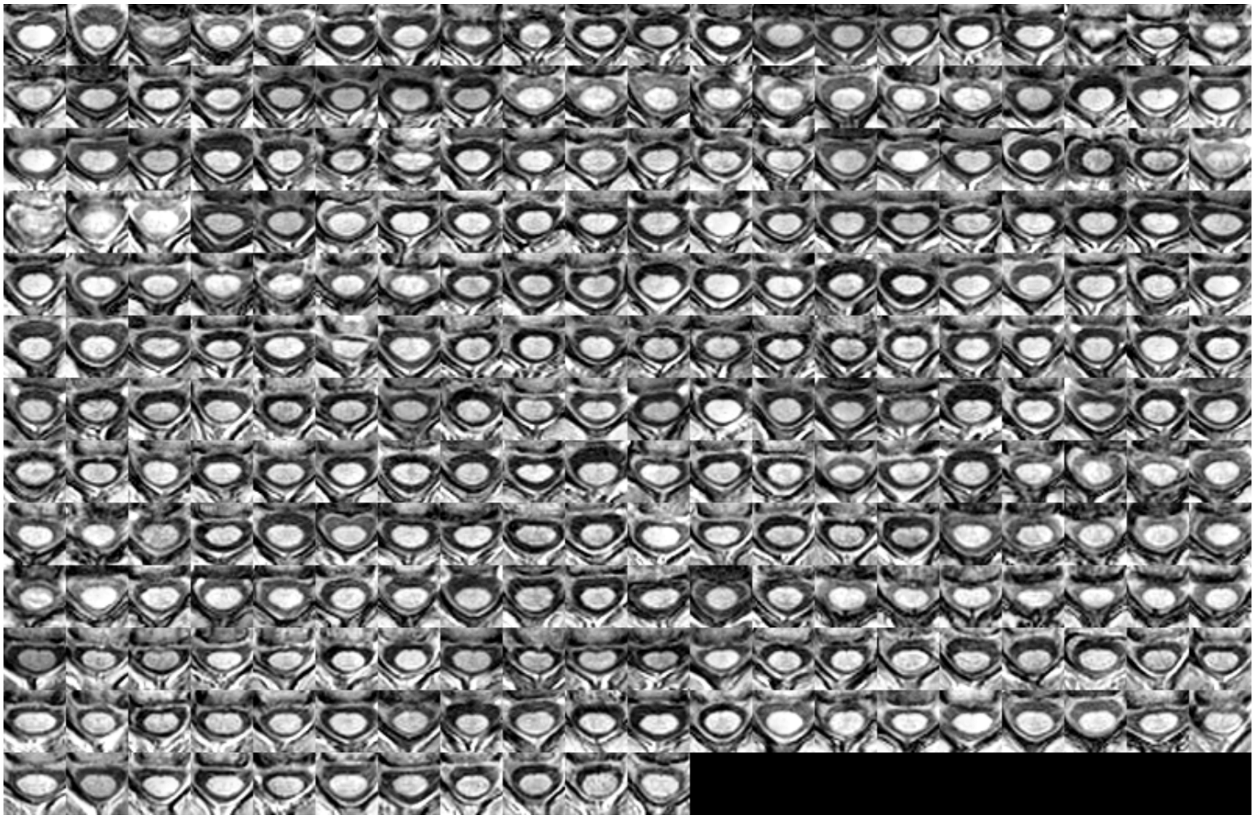

Figure S4. *GRE-T1w axial images (mid-slice) of individual participants from the multi-subject database.*

Example DWI data

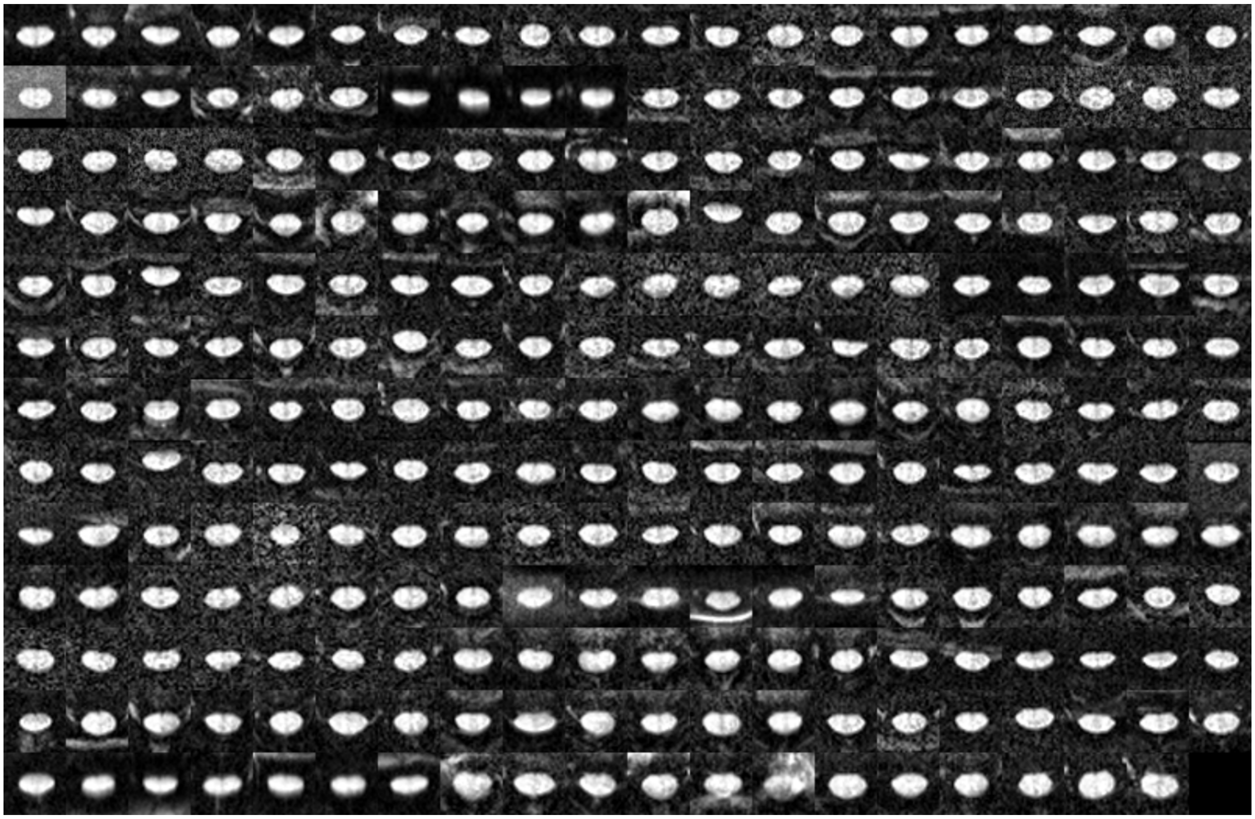

Figure S5. *Mean DWI axial images (mid-slice) of individual participants from the multi-subject database.*
